# A Chemical Counterpart to the Resolution Step of Nature’s Intein-Mediated Protein Splicing

**DOI:** 10.1021/acschembio.3c00590

**Published:** 2023-12-14

**Authors:** Balamurugan Dhayalan, Stephen B. H. Kent, Ingrid Fetter-Pruneda

**Affiliations:** † Department of Chemistry, University of Chicago, 929 East 57th Street, Chicago, Illinois 60637, United States; ‡ Laboratory of Social Evolution and Behavior, The Rockefeller University, 1230 York Avenue, New York, New York 10065, United States

## Abstract

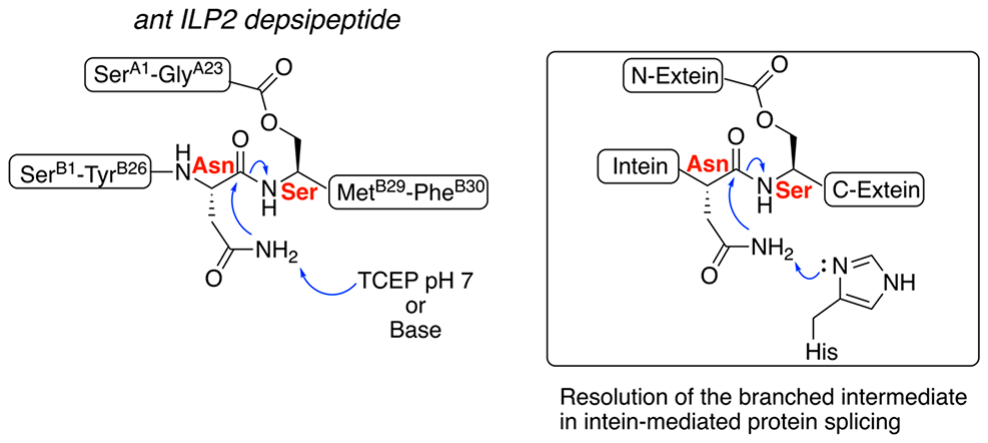

In the course of an attempted total chemical synthesis of the ant insulin-like peptide-2 (ILP2) protein molecule, specific cleavage of a backbone peptide bond in a branched ester-linked polypeptide chain with concomitant peptide splicing was observed. The side reaction was investigated in model compounds. Here, we postulate a chemical mechanism for this novel polypeptide backbone cleavage reaction as a chemical counterpart to the resolution step of biochemical intein-mediated protein splicing.

Insulin and insulin-like peptide (ILP) signaling is a highly evolutionarily conserved pathway that is involved in metabolism regulation throughout the animal kingdom. Insects have ILPs that are expressed in the brain and other tissues such as the fat body, midgut, and salivary glands.^1^ Studies in *Drosophila melanogaster* have revealed that ILPs play crucial roles in regulating development, longevity, metabolism, and female reproduction.^2^

In social insects, the insulin/insulin-like growth factor signaling (IIS) pathway is considered a key player in governing two important phenomena: developmental polyphenism (where a single genotype generates distinct phenotypes, like queens and workers, in response to environmental cues)^3^ and polyethism (the age-related division of labor within social insect colonies).^4^ In ants, the expression of the *insulin-like peptide 2* (*ilp2*) gene is consistently higher in the brains of reproductive individuals, even among distantly related species. Additionally, the expression of *ilp2* is influenced by the social environment, and pharmacologically increasing its levels leads to an increase in ovarian activity.^5^ This suggests that the insulin signaling pathway might have played a pivotal role in the evolution of eusociality, particularly in the reproductive division of labor in ants.^5^ In addition, the expression of insulin is associated with increased longevity and fertility in ants.^6^ Given its biological significance, we embarked on a project to chemically synthesize ILP2 to facilitate its use in pharmacological experiments involving ants.

By analogy with other insulin-like proteins, ant insulin-like peptide 2 (ILP2) is predicted to consist of two peptide chains and to contain three disulfide bonds.^7−9^ The predicted amino acid sequence and disulfide bonds of ant ILP2 were based on the genomic and transcriptomic sequence of the ILP2 polypeptide chain from the clonal raider ant *Ooceraea biroi*.^10^ In the predicted covalent structure of the mature folded ant ILP2 protein molecule, the peptide chains are connected by two interchain disulfide bonds. There is also one intrachain disulfide bond (Figure 1A).

**Figure 1:**
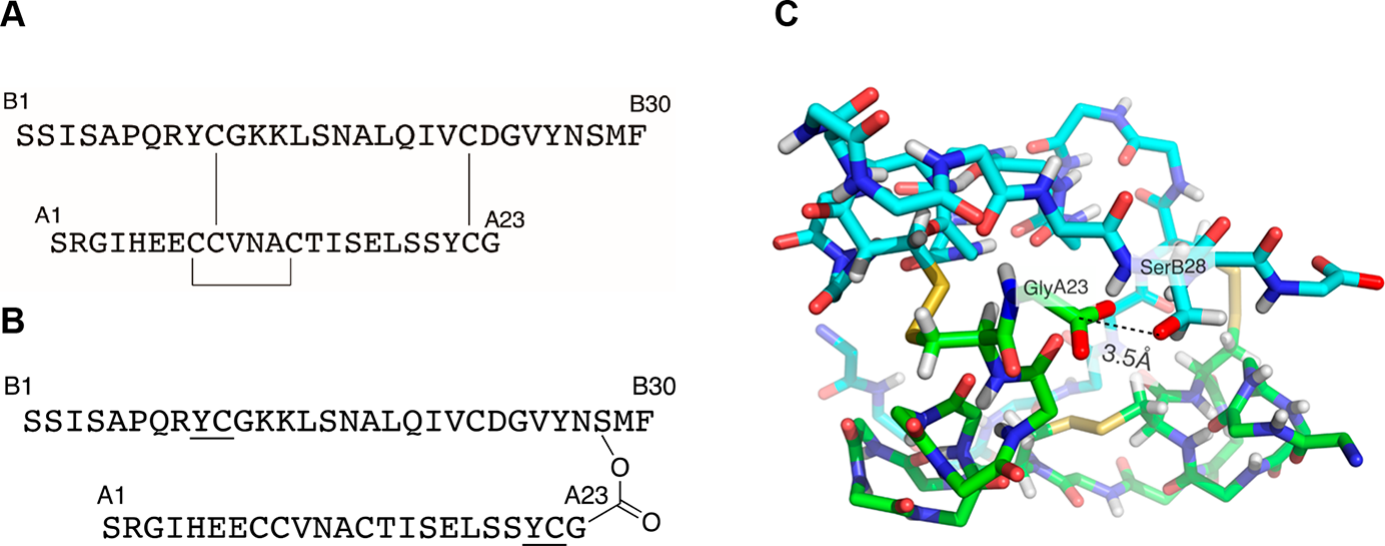
Ant ILP2. (A) Predicted covalent structure of the ant ILP2 protein. The A chain has 23 amino acid residues, and the B-chain has 30 amino acids. Predicted disulfide bonds are labeled as connecting lines between the involved cysteine residues. (B) Designed ester-linked polypeptide chain precursor to ant ILP2. Tyr^A21^-Cys^A22^ and Tyr^B9^-Cys^B10^. Ligation sites are underlined. (C) Ant ILP2 homology model based on the structure of human DKP insulin (PDB code 2JUV). The A chain alpha-COOH moiety of Gly^A23^ is in close proximity to the side chain −OH of Ser^B28^. Colors: A chain, green; B chain, cyan; disulfide bonds, yellow.

We set out to prepare ILP2 by a total chemical synthesis. Chemical synthesis of the two-chain protein molecules from the insulin superfamily is challenging. Simply mixing the A and B peptide chains under conventional folding conditions gives very low yields of correctly folded, disulfide-linked protein.^11−14^ Since Sanger’s original report of the covalent structure of bovine insulin,^15^ many different approaches to the total chemical synthesis of insulin superfamily proteins have been devised.^16,17^ In 2010, we described a chemical analog of the proinsulin precursor polypeptide, a branched ester-linked synthetic polypeptide chain that folded efficiently at physiological pH to generate an “ester insulin” protein molecule containing the three native disulfide bonds of insulin.^18^ Simple saponification at reduced temperature was used to convert ester insulin to fully active human insulin, in good yield.^19^ Here, we devised an analogous approach to the total chemical synthesis of the predicted ant ILP2 protein. Homology modeling of the folded structure of ant ILP2, based on the known structure of the human DKP insulin protein molecule,^20^ suggested that in the predicted ant ILP2 protein molecule the alpha-COOH of Gly^A23^ at the C-terminus of the A chain would be in close proximity to the side chain −OH of Ser^B28^ in the B chain (Figure 1C).

Based on this observation from the homology model, we designed a polypeptide chain (shown in Figure 1B to highlight its relatedness to the target ant ILP2 molecule) containing an ester linkage joining the Ser^B28^ side chain −OH to Gly^A23-α^COOH as a key intermediate for the total synthesis of ant ILP2. This branched depsipeptide would be expected to fold efficiently and could then be saponified to give the mature ant ILP2 protein molecule containing the correct disulfide bonds.

A convergent route to the ester-linked 53 residue polypeptide chain from three synthetic peptide segments is shown in Scheme 1. The synthesis makes use of native chemical ligation of unprotected peptide segments.^21^ Peptide segments Ser^B1^-Tyr^B9^ thioester (**1**), Ser^A1^-Tyr^A21^ thioester (**2**), and Thz^B10^-Ser^B28^(OGly^A23^-Cys^A22^)-Phe^B30^ (**3**) were prepared by stepwise solid phase peptide synthesis (SPPS; Supporting Information, Figures S5–S7). An orthogonally protected ester-linked dipeptide Fmoc-Ser[O(Boc-Gly)]–OH (inset, Scheme 1) was prepared and used in the synthesis of Thz^B10^-Ser^B28^(OGly^A23^-Cys^A22^)-Phe^B30^ (**3**; Supporting Information, Figures S1–S4).

**Scheme 1:**
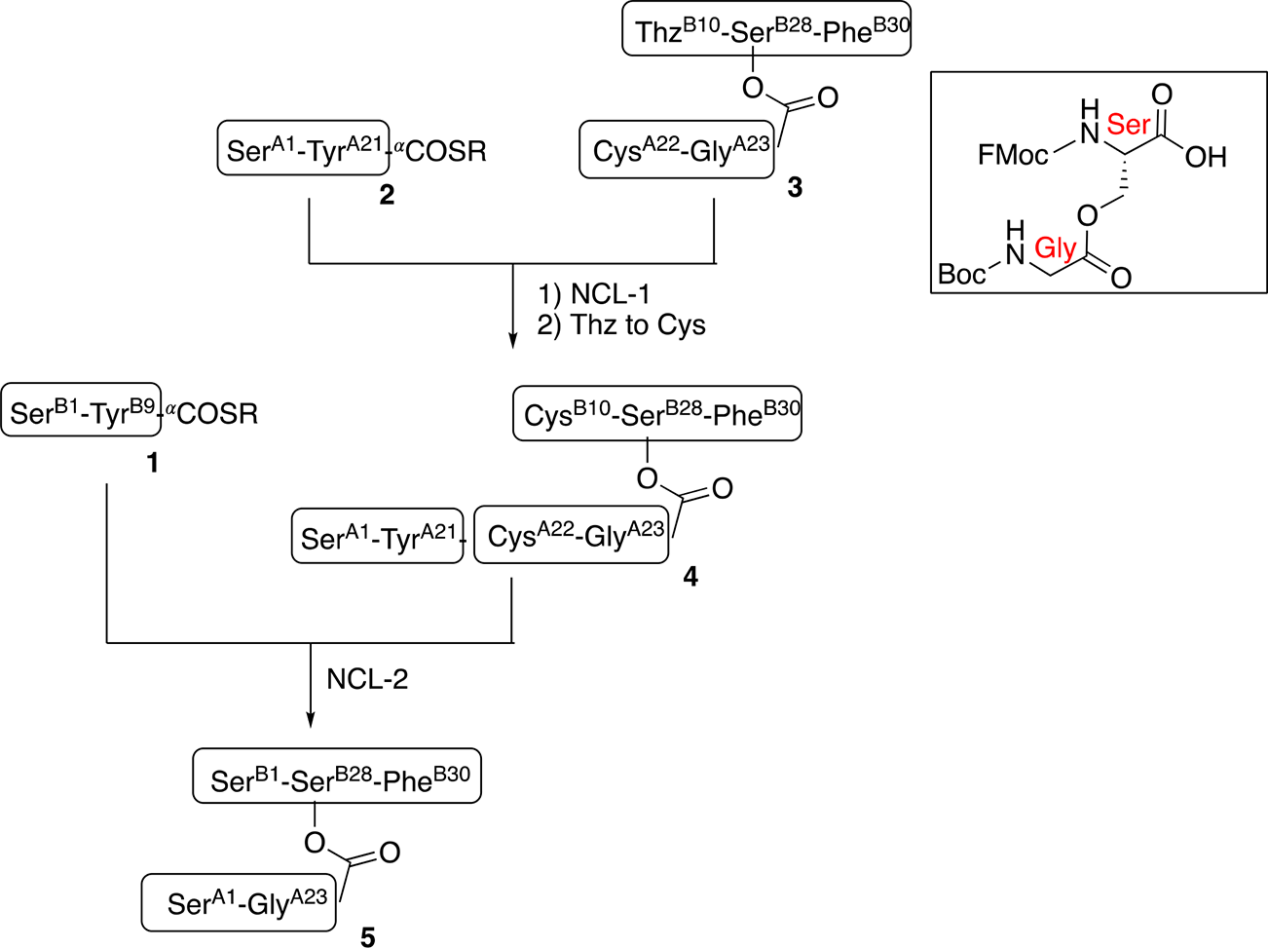
Proposed Convergent Synthesis of an Ester-Linked Depsipeptide Precursor to Ant ILP2 by Native Chemical Ligation of Three Peptide Segments The orthogonally protected ester-linked dipeptide Fmoc-Ser[O(Boc-Gly)]–OH, used in the synthesis of peptide segment **3**, is shown in the inset. NCL, native chemical ligation;^21^ Thz, L-1,3-thiazolidine-4-carboxylic acid.

Synthesis of the branched ester linked peptide according to Scheme 1 was carried out as a series of “one pot” reactions, without purification of intermediate products.^22^ Native chemical ligation reactions were performed in aqueous 6 M GuHCl/0.1 M NaH_2_PO_4_ (Pi) buffer at pH 7.0 containing 200 mM 4-mercaptophenylacetic acid (MPAA). For the first ligation, the N-terminal Cys^B10^ of thioester-containing peptide segment **3** was rendered unreactive as a thiazolidine (Thz−) moiety. After ligation, the Thz was converted to reactive Cys by treatment with MeONH_2_·HCL at pH 4. The solution was readjusted to pH 7, and the second ligation was carried out by the addition of Ser^B1^-Tyr^B9^ thioester (**1**).

After the first native chemical ligation reaction, in addition to expected product **6**, we observed the formation of byproducts whose masses were consistent with cleavage of the expected ligation product at the Asn^B27^-Ser^B28^ peptide bond, to give fragments Thz^B10^-Asn^B27^ and Ser^A1^-Gly^A23^-Ser^B28^-Phe^B30^ (Supporting Information, Figure S8). The observed mass (−18 Da) of the Thz^B10^-Asn^B27^ fragment showed that C-terminal Asn^B27^ had been cyclized to the succinimide. Furthermore, the initial ester-linked Ser^B28^[O(Gly^A23^- Ser^A1^)]-Phe^B30^ cleavage fragment had undergone an O-to-N acyl rearrangement, to give the amide-linked chimeric Ser^A1^-Gly^A23^-Ser^B28^-Phe^B30^ fragment, as shown by its resistance to saponification at pH 12 (Supporting Information, Figure S11).

After the second native chemical ligation, related products **5b** and **6b** were formed (Figure 2) by cleavage of final ligation product **5** at the same Asn^B27^-Ser^B28^ peptide bond, with concomitant formation of the succinimide form of the C-terminal Asn^B27^. Cleavage of the Asn^B27^-Ser^B28^ peptide bond was also observed under the pH 7.6 folding conditions used to convert the ester-linked polypeptide **5** to the disulfide containing ester-linked ILP2 protein (Figure S12) and under the pH 12 LiOH saponification conditions used to generate the mature two-chain ant ILP2 protein (Figure S13).

**Figure 2:**
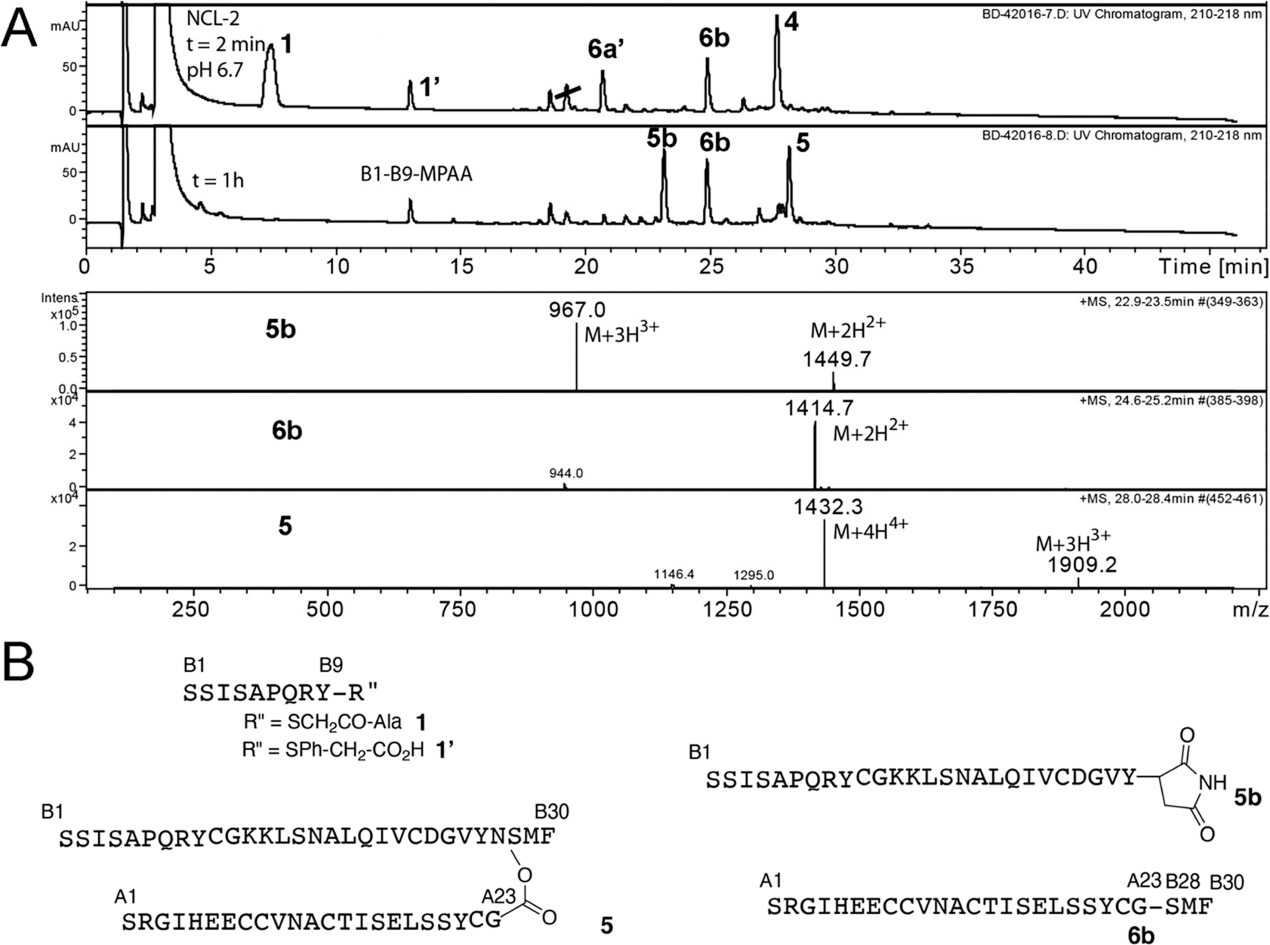
LCMS data after the second NCL reaction. Peak **5** is the desired depsipeptide product; cleavage of the depsipeptide chain at the Asn^B27^-Ser^B28^ peptide bond gave Cys^B10^-Asn^B27^(succinimide) (**5b**) and rearranged Ser^A1^-Gly^A23^-Ser^B28^-Phe^B30^ (**6b**).

The complex product mixtures resulting at every stage of the synthesis from cleavage at the Asn^B27^-Ser^B28^ peptide bond, with concomitant splicing of segments of the A and B chain peptides, caused us to abandon this route to the total chemical synthesis of ant IPL2.

Instead, we set out to understand this unanticipated side reaction. In order to establish a basis for a mechanistic hypothesis for this novel peptide chain cleavage reaction, we systematically investigated the stability of model peptides containing the −Asn–Ser(OGly)– moiety. Peptide segment Thz^B10^-Ser^B28^[O(Gly^A23^-Cys^A22^)]-Phe^B30^ was subjected to treatment at pH 7.0 in the presence and absence of TCEP. In the presence of TCEP the cleavage reaction was prominent. Next, we prepared the model peptide Ac-Phe-Arg-Ala-Asn-Ser(OAc)-Phe-Arg-Ala containing an acetyl ester (OAc) on the Ser side chain OH (Figure S14). Surprisingly, it was completely stable after 24 h of treatment with 50 mM TCEP·HCl at a pH of 7.0 (Supporting Information, Figure S15).

Additional model studies were designed to more faithfully mimic the original Thz^B10^-Ser^B28^[O(Gly^A23^-Cys^A22^)]-Phe^B30^ peptide, with variations of potential H-bond donors on the Ser-side chain (Figures S16 and S17). The peptide Ac-Phe-Arg-Ala-Asn-Ser(O-Gly)-Phe-Arg-Ala underwent similar extents of cleavage with (54%) or without (60%) TCEP at a pH of 7.0. The peptide Ac-Phe-Arg-Ala-Asn-Ser(O-Gly-Ac)-Phe-Arg-Ala underwent a similar cleavage reaction, but the rate of cleavage was somewhat slower when TCEP was not present (22%) versus 66% cleavage in the presence of TCEP (Figure S18).

The pH dependence of cleavage was investigated for both Ac-Phe-Arg-Ala-Asn-Ser(O-Gly)-Phe-Arg-Ala and Ac-Phe-Arg-Ala-Asn-Ser(O-Gly-Ac)-Phe-Arg-Ala. The conditions were pH 5.5 and pH 8.0, with TCEP [6 M GuHCl, 0.1 M Pi buffer, 50 mM TCEP] or without TCEP [6 M GuHCl, 0.1 M Pi buffer]. After 24 h of reaction at a pH of 5.5, cleavage was <8% for both model peptides (Figure S19). At a pH of 8.0, the cleavage reaction was accelerated with or without TCEP, and 84–95% cleavage was seen within 5 h (Figure S20).

The results of these model studies are summarized in Table 1. Ac-Phe-Arg-Ala-Asn-Ser(O-*COCH*_***3***_)-Phe-Arg-Ala did not undergo cleavage under any of the conditions examined.

**Table 1:** Model Studies of the Cleavage Reaction

pH	time	condition	Ac-Phe-Arg-Ala-Asn-Ser(O-Gly)-Phe-Arg-Ala	Ac-Phe-Arg-Ala-Asn-Ser(O-Gly-Ac)-Phe-Arg-Ala
5.5	24 h	no TCEP	8%	4%
		50 mM TCEP	8%	5%
7.0	24 h	NO TCEP	54%	22%
		50 mM TCEP	60%	66%
8.0	5 h	NO TCEP	88%	95%
		50 mM TCEP	84%	89%

Considered together, these data show that the cleavage reaction is base catalyzed and that a neighboring amide group (as in Ac-Gly-, CH_3_CONHCH_2_CO-, or peptidyl-Gly) or a free amino group (as in Gly, H_2_NCH_2_CO- at a pH of 8) is required for the cleavage reaction. For −Asn–Ser(OGly-peptidyl)– under native chemical ligation reaction conditions at a pH of 7, TCEP serves as the base.

Based on these observations, a plausible mechanism for the chemical cleavage reaction at −Asn–Ser(O-peptidyl)– is shown in Scheme 2. Similar to the resolution step of protein splicing, the thermodynamic driving force for the cleavage of the −Asn-Ser(O-peptidyl)– peptide bond is base-catalyzed formation of a succinimide at the postcleavage C-terminal asparagine residue. The other, initially ester-linked, Ser(O-peptidyl)– fragment undergoes a spontaneous O-to-N acyl shift to give the more stable amide-linked peptidyl-Ser– product in which the peptide sequence originally attached to the side chain hydroxyl of the Ser residue becomes attached through a peptide bond to the C-terminal cleavage fragment, forming a chimeric peptide chain product.

**Scheme 2:**
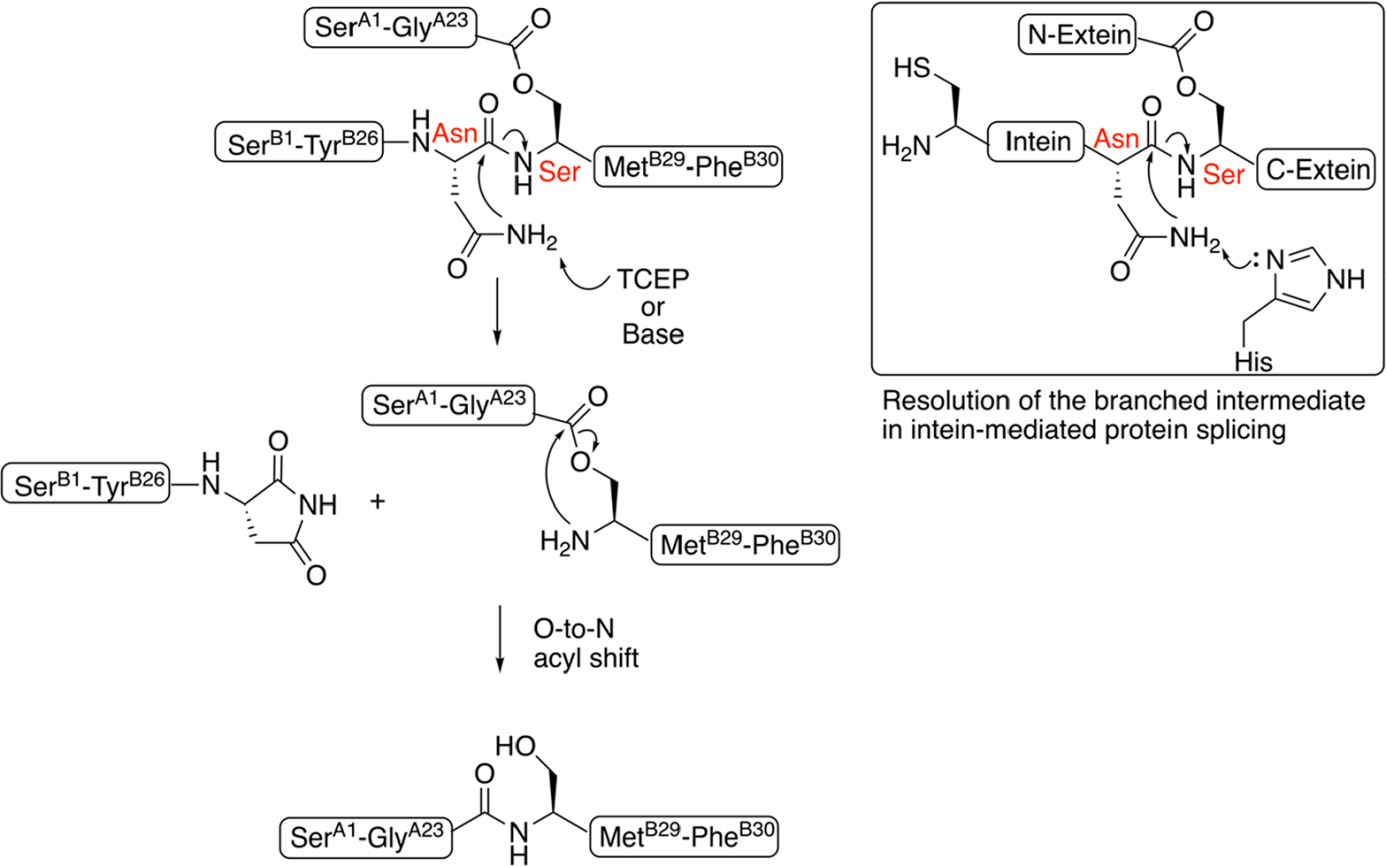
Proposed Reaction Mechanism for the Chemical Cleavage of the Ant ILP2 Depsipeptide (Inset, on the Right, Similarities of Positioning of Amino Acid Side Chains and Local Structure around the Cleavage Site Are Evident in Comparison to Branched Intermediate Resolution Step of the Intein-Mediated Protein Splicing Reaction)

The mechanism proposed for the chemical cleavage-peptide splicing reaction reported in this work is in concordance with the mechanism of the resolution step in intein-mediated protein splicing proposed by Muir et al., with respect to the necessity of a side chain H-bond and requirement of a base catalyst for the reaction.^23^ Interestingly, in contrast to our results, Wasmuth et al. found no requirement for an amine or amide moiety in the native intein-mediated addition of nonpeptide moieties to the C-extein polypeptide.^24^ We conclude that the novel −Asn-Ser(O-peptidyl)– cleavage/splicing side reaction that we encountered is a chemical counterpart to the resolution step of intein-mediated biochemical protein splicing. Access to a readily accessible peptide model system will permit systematic studies to elucidate further details of the mechanism of the resolution step of nature’s intein-mediated protein splicing reaction.

## Methods

### Preparation of Fmoc-Ser[O(BocGly)]–OH

2-Bromoacetophenone (2.73 g, 13.69 mmol) was dissolved in 20 mL of DMF and added to the mixture of Fmoc-Ser(OtBu)–OH (5 g, 13.04 mmol) and K_2_CO_3_ (3.6 g, 26.08 mmol) in 10 mL of DMF. The reaction was stirred for 4 h. TLC showed complete consumption of the starting material, diluted with water (300 mL) and extracted with ethyl acetate (3 × 100 mL). The organic layer was dried on Na_2_SO_4_, filtered, evaporated under reduced pressure, and lyophilized to give the product Fmoc-Ser(OtBu)-OPac as a pale-yellow solid (6.3 g, 13.2 mmol, 96.3%). LCMS (ESI) data, obsd.: 524.2 ± 0.1 Da. Calculated (M + Na): 524.2 Da.

Fmoc-Ser(OtBu)-OPac (6.0 g, 12.56 mmol) was treated with 95:5 TFA/water (50 mL) for 2 h. After TLC showed the completion of the reaction, excess solvent was evaporated under reduced pressure. The viscous liquid was taken up in 1:1 *v*/*v* acetonitrile/water and lyophilized to give 5.5 g of the crude product as a pale-yellow solid. The product mixture also contained ∼15% of Fmoc-Ser(OCOCF_3_)-OPac. Without further purification, the product was used in the next step. LCMS (ESI) data obsd.: 468.1 ± 0.1 Da> Calculated (M + Na): 468.2 Da. Crude Fmoc-Ser(OH)-OPac (5.5 g, 12.35 mmol), Boc-Gly-OH (4.3 g, 24.7 mmol), EDCI·HCl (4.73 g, 24.7 mmol), and DMAP (302 mg, 2.47 mmol) were dissolved in 50 mL of DCM and stirred for 2 h at RT under an argon atmosphere. After TLC indicated completion of the reaction, all of the volatiles were evaporated under reduced pressure, and the residue was taken up in 200 mL of ethyl acetate and washed with 1 M ammonium chloride. The organic layer was dried in Na_2_SO_4_ and evaporated. The resulting viscous liquid was loaded onto a silica gel column and eluted with 10–40% ethyl acetate/hexanes. Evaporation of fractions selected by TLC gave the desired product Fmoc-Ser[O(Boc-Gly)]-OPac as a semisolid (6.5 g, 10.8 mmol, 87.3%). LCMS (ESI) data obsd.: 625.2 ± 0.2 Da. Calculated (M + Na): 625.2 Da.

Zinc dust (2 g) was added to dissolved Fmoc-Ser[O(Boc-Gly)]-OPac ester (6.5 g, 10.78 mmol) stirred in 75 mL of acetic acid. A second portion of zinc dust (1 g) was added after 2 h, and the reaction mixture was stirred for an additional 2 h, after which time all the starting materials were consumed (TLC). The reaction mixture was filtered on a 10 μ fritted funnel and washed with ethyl acetate. Filtrates were combined and evaporated to dryness. The crude product was dissolved in a minimal amount of DCM, loaded onto a silica gel column, and eluted using 1 to 5% methanol in DCM as the mobile phase. Selected fractions, based on TLC, were evaporated, and the resulting semisolid after trituration with hexane and drying under a high vacuum gave the title compound Fmoc-Ser[O(BocGly)]–OH as a fluffy solid (3.9 g, 8.05 mmol, 74.7%). LCMS (ESI) data obsd.: 507.3 ± 0.1 Da. Calculated: (M + Na), 507.2 Da.

### Synthesis of Peptides **1**, **2**, and **3**

Stepwise “in situ neutralization” Boc chemistry SPPS was used as follows. Starting resin: Boc-Xaa-OCH_2_-Pam-resin (0.2 mmol; where Xaa = Ala or Phe). Deprotection and washing: 10 mL TFA flow (10 s), 2 × 5 mL TFA batch (1 min each), DMF flow (30 s). In situ neutralization and simultaneous coupling: Boc-AA-OH (1.1 mmol) was dissolved in 0.5 M HBTU (1.0 mmol, 2 mL), and DIEA (1.5 mmol, 261 μL) was added to the mixture. After 30 s of activation, the solution was added to the peptide-resin and reacted for 12 min. After assembly of the target sequence by SPPS, the final N^α^-Boc group was removed, and the peptide was cleaved from the resin with simultaneous removal of side-chain protecting groups using 5% p-cresol/95% HF at 0 °C for 1 h. After the evaporation of HF at 0 °C, the resulting residue was treated with ice-cold ether. The precipitated peptide was recovered by filtration and washed with ice-cold ether. Crude peptide was dissolved directly in 6 M GuHCl, acidified, and purified using preparative HPLC on a C18 column (9.4 × 250 mm) to give each target peptide. Full details can be found in the Supporting Information.

### Synthesis of Ant ILP2 Full-Length Depsipeptide

Based on the preliminary results described in the Supporting Information, synthesis of the ant ILP2 full-length depsipeptide was repeated under the following modified conditions. The key modifications were as follows: MPAA concentration increased from 100 to 200 mM during the first ligation (in 2 h, ligation was complete), and the MeONH_2_·HCl concentration was increased from 200 to 400 mM for the conversion of Thz to Cys (complete conversion within 5 h). Detailed conditions were as follows.

#### First Native Chemical Ligation

Ser^A1^-Tyr^A21^-SCH_2_CO-Arg_4_-Ala (**2**, 5.0 mg, 1.544 μmol, 3.1 mM; note: with His-DNP) and Thz^B10^-Ser^B28^[O(Gly^A23^-Cys^A22^)]-Phe^B30^ (**3**, 3.2 mg, 1.287 μmol, 2.57 mM) were dissolved in aqueous 6 M guanidine·HCl, containing 200 mM MPAA, buffered by 0.1 M Pi at a pH of 7.0. After 2 h, 50 mM TCEP·HCl (7.2 mg) was added for 5 min.

#### Thz to Cys Conversion

MeONH_2_·HCl (16.7 mg, 400 mM) was added, the pH was adjusted to 4.0. The reaction continued for 5 h.

#### Second Native Chemical Ligation

Ser^B1^-Tyr^B9^-SCH_2_CO-Ala (**1**, 2.2 mg, 1.93 μmol) and 50 mM MPAA (3.36 mg) was added and the reaction mixture adjusted to pH 6.7; ligation was complete within 1 h (Figure S9).

### Studies of Parameters Affecting the Chain Cleavage Reaction

Model peptides were prepared using Fmoc chemistry SPPS. Side-chain protecting groups for Fmoc-amino acids used were Arg(Pbf), Asn(Trt), and Ser(tBu). The Fmoc chemistry stepwise SPPS protocol used was as follows: scale, 0.1 mg of H-Ala-O-2-chlorotrityl-(S-DVB) resin; DMF washes, 10 s flow, 1 × 1 min batch; Fmoc-AA (0.55 mmol) dissolved in 0.5 M HBTU in DMF (1 mL, 0.5 mmol); 0.75 mmol DIEA (131 μL) added; after 30 s of activation, the solution was added to the peptide-resin; coupling, 30 min. N^α^Fmoc removal: 20% v/v piperidine/DMF 2 × 5 min batch treatments. The product peptide was cleaved from the -Ala-2-chlorotrityl-(S-DVB) resin and simultaneously deprotected by subjecting it to TFA/TIPS/water/EDT (95:2:2:1 v/v) conditions at ambient temperature. The crude peptides were purified by preparative HPLC.

## Supporting Information Available

Experimental details of peptide syntheses, native chemical ligation reactions, studies on model peptides (PDF)

## References

[ref1] Chowański S.; Walkowiak-Nowicka K.; Winkiel M.; Marciniak P.; Urbański A.; Pacholska-Bogalska J. Insulin-like Peptides and Cross-Talk with Other Factors in the Regulation of Insect Metabolism. Front. Physiol. 2021, 12, 973 10.3389/fphys.2021.701203. PMC8276055 34267679

[ref2] Wu Q.; Brown M. R. Signaling and Function of Insulin-like Peptides in Insects. Annu. Rev. Entomol 2006, 51, 1–24. 10.1146/annurev.ento.51.110104.151011. 16332201

[ref3] Corona M.; Libbrecht R.; Wheeler D. E. Molecular Mechanisms of Phenotypic Plasticity in Social Insects. Curr. Opin. Insect Sci. 2016, 13, 55–60. 10.1016/j.cois.2015.12.003. 27436553

[ref4] Ament S. A.; Corona M.; Pollock H. S.; Robinson G. E. Insulin Signaling Is Involved in the Regulation of Worker Division of Labor in Honey Bee Colonies. Proc. Natl. Acad. Sci. U. S. A. 2008, 105 (11), 4226–4231. 10.1073/pnas.0800630105. 18337502 PMC2393790

[ref5] Chandra V.; Fetter-Pruneda I.; Oxley P. R.; Ritger A. L.; McKenzie S. K.; Libbrecht R.; Kronauer D. J. C. Social Regulation of Insulin Signaling and the Evolution of Eusociality in Ants. Science 2018, 361 (6400), 398–402. 10.1126/science.aar5723. 30049879 PMC6178808

[ref6] Yan H.; Opachaloemphan C.; Carmona-Aldana F.; Mancini G.; Mlejnek J.; Descostes N.; Sieriebriennikov B.; Leibholz A.; Zhou X.; Ding L.; Traficante M.; Desplan C.; Reinberg D. Insulin Signaling in the Long-Lived Reproductive Caste of Ants. Science 2022, 377 (6610), 1092–1099. 10.1126/science.abm8767. 36048960 PMC9526546

[ref7] Sanger F. Chemistry of Insulin: Determination of the Structure of Insulin Opens the Way to Greater Understanding of Life Processes. Science 1959, 129 (3359), 1340–1344. 10.1126/science.129.3359.1340. 13658959

[ref8] Nicol D. S. H. W.; Smith L. F. Amino-Acid Sequence of Human Insulin. Nature 1960, 187 (4736), 483–485. 10.1038/187483a0. 14426955

[ref9] Ryle A. P.; Sanger F.; Smith L. F.; Kitai R. The Disulphide Bonds of Insulin. Biochem. J. 1955, 60 (4), 541–556. 10.1042/bj0600541. 13249947 PMC1216151

[ref10] Oxley P. R.; Ji L.; Fetter-Pruneda I.; McKenzie S. K.; Li C.; Hu H.; Zhang G.; Kronauer D. J. C. The Genome of the Clonal Raider Ant Cerapachys Biroi. Curr. Biol. 2014, 24 (4), 451–458. 10.1016/j.cub.2014.01.018. 24508170 PMC3961065

[ref11] Marglin B.; Merrifield R. B. The Synthesis of Bovine Insulin by the Solid Phase Method ^1^. J. Am. Chem. Soc. 1966, 88 (21), 5051–5052. 10.1021/ja00973a068. 5978833

[ref12] Katsoyannis P. G.; Tometsko A.; Zalut C. Insulin Peptides. XII. Human Insulin Generation by Combination of Synthetic A and B Chains ^1^. J. Am. Chem. Soc. 1966, 88 (1), 166–167. 10.1021/ja00953a033. 5900390

[ref13] Meienhofer J.; Schnabel E.; Bremer H.; Brinkhoff O.; Zabel R.; Sroka W.; Klostermeyer H.; Brandenburg D.; Okuda T.; Zahn H. Notizen: Synthese Der Insulinketten Und Ihre Kombination Zu Insulinaktiven Präparaten. Z. Für Naturforschung B 1963, 18 (12), 1120–1120. 10.1515/znb-1963-1223. 14117584

[ref14] Du Y.-C.; Jiang R.-Q.; Tsou C.-L. Conditions for Successful Resynthesis of Insulin from Its Glycyl and Phenylalanyl Chains. Sci. Sin. 1965, 14 (2), 229.

[ref15] Sanger F.; Smith L. The Structure of Insulin. Endeavour 1957, 16 (61), 48–53.

[ref16] Mayer J. P.; Zhang F.; DiMarchi R. D. Insulin Structure and Function. Biopolymers 2007, 88 (5), 687–713. 10.1002/bip.20734. 17410596

[ref17] Hossain M. A.; Wade J. D. Novel Methods for the Chemical Synthesis of Insulin Superfamily Peptides and of Analogues Containing Disulfide Isosteres. Acc. Chem. Res. 2017, 50 (9), 2116–2127. 10.1021/acs.accounts.7b00288. 28829564

[ref18] Sohma Y.; Hua Q.-X.; Whittaker J.; Weiss M. A.; Kent S. B. H. Design and Folding of [GluA4(OβThrB30)]Insulin (“Ester Insulin”): A Minimal Proinsulin Surrogate That Can Be Chemically Converted into Human Insulin. Angew. Chem., Int. Ed. 2010, 49 (32), 5489–5493. 10.1002/anie.201001151. PMC3311283 20509131

[ref19] Dhayalan B.; Fitzpatrick A.; Mandal K.; Whittaker J.; Weiss M. A.; Tokmakoff A.; Kent S. B. H. Efficient Total Chemical Synthesis of ^13^ C= ^18^ O Isotopomers of Human Insulin for Isotope-Edited FTIR. ChemBioChem. 2016, 17 (5), 415–420. 10.1002/cbic.201500601. 26715336 PMC5477233

[ref20] Huang K.; Chan S. J.; Hua Q.; Chu Y.-C.; Wang R.; Klaproth B.; Jia W.; Whittaker J.; De Meyts P.; Nakagawa S. H.; Steiner D. F.; Katsoyannis P. G.; Weiss M. A. The A-Chain of Insulin Contacts the Insert Domain of the Insulin Receptor. J. Biol. Chem. 2007, 282 (48), 35337–35349. 10.1074/jbc.M705996200. 17884811

[ref21] Dawson P. E.; Muir T. W.; Clark-Lewis I.; Kent S. B. H. Synthesis of Proteins by Native Chemical Ligation. Science 1994, 266 (5186), 776–779. 10.1126/science.7973629. 7973629

[ref22] Bang D.; Kent S. B. H. A One-Pot Total Synthesis of Crambin. Angew. Chem., Int. Ed. 2004, 43 (19), 2534–2538. 10.1002/anie.200353540. 15127445

[ref23] Liu Z.; Frutos S.; Bick M. J.; Vila-Perelló M.; Debelouchina G. T.; Darst S. A.; Muir T. W. Structure of the Branched Intermediate in Protein Splicing. Proc. Natl. Acad. Sci. U. S. A. 2014, 111 (23), 8422–8427. 10.1073/pnas.1402942111. 24778214 PMC4060664

[ref24] Wasmuth A.; Ludwig C.; Mootz H. D. Structure–Activity Studies on the Upstream Splice Junction of a Semisynthetic Intein. Bioorg. Med. Chem. 2013, 21 (12), 3495–3503. 10.1016/j.bmc.2013.03.065. 23618706

