# Supplementary material for: A Chemical Counterpart to the Resolution Step of Nature’s Intein-Mediated Protein Splicing
Source: ACS Chem Biol. 2023 Dec 14;19(1):9–14. doi: 10.1021/acschembio.3c00590 (PMC10804359; doi:10.1021/acschembio.3c00590)
Supplement: Supplementary file 1 — cb3c00590_si_001.pdf [file cb3c00590_si_001.pdf]

Supporting Information

A Chemical Counterpart to the Resolution Step of Nature's Intein-Mediated Protein Splicing

Balamurugan Dhayalan,[1,^] Stephen B. H. Kent,[1] and Ingrid Fetter-Pruneda*[2,#]*

[1] Department of Chemistry, University of Chicago, 929 East 57th Street, Chicago, IL 60637, USA.

[^] Current address: Department of Biochemistry and Molecular Biology, Indiana University School of Medicine, 635 Barnhill Rd, Indianapolis, IN 46202, USA.

[2] Laboratory of Social Evolution and Behavior, The Rockefeller University, 1230 York Avenue, New York, NY 10065, USA.

[#] Current address: Departamento de Biología Celular y Fisiología, Instituto de Investigaciones Biomédicas, Universidad Nacional Autónoma de México, Mexico City 04510, Mexico. *e-mail: skent@uchicago.edu, ifetter@iibiomedicas.unam.mx

Contents **Page No.**

1. Chemicals and reagents……………………………………………………………S3

2. Analytical LC-MS…………………………………………………………………S3

3. Preparative Reverse-phase HPLC purifications……………………….…………...S4

4. Synthesis of Fmoc-Ser[O(Boc-Gly)]-OH dipeptide………………………....S4-S8

5. Synthesis of peptide segments **1-3**………………………………………….S9-S12

6. Synthesis of ant insulin full length peptide **5**………..……………….……S12-S16

7. Stability of ester bond under LiOH saponification conditions ……….………..S17

8. Folding of ant insulin full length peptide **5**………………………………..S18-S19

9. Synthesis of Ac-Phe-Arg-Ala-Asn-Ser(OAc)-Phe-Arg-Ala………………S20-S21

10. Stability studies of Ac-Phe-Arg-Ala-Asn-Ser(OAc)-Phe-Arg-Ala…………….S22

11. Synthesis of Ac-Phe-Arg-Ala-Asn-Ser(O-Gly)-Phe-Arg-Ala ……………S23-S24

12. Synthesis of Ac-Phe-Arg-Ala-Asn-Ser(O-Gly-Ac)-Phe-Arg-Ala ………..S25-S26

13. Studies on the stability of Ac-Phe-Arg-Ala-Asn-Ser(O-Gly)-Phe-Arg-Ala and Ac-Phe-Arg-Ala-Asn-Ser(O-Gly-Ac)-Phe-Arg-Ala at different pH values ….S27-S29

14. Stability of peptide Thz^{B10}-Ser^{B28}[O(Gly^{A23}-Cys^{A22})]-Phe^{B30} at pH 7 in the presence and absence of TCEP…………………………………………………………...S30

15. References………………………………………………………………………S31

1. Chemicals and Reagents

All amino acids used were of the L-configuration, and achiral glycine. Boc-amino acids, Fmoc amino acids (Peptide Institute, Osaka) and N,N,N′,N′-Tetramethyl-O-(1H-benzotriazol-1-yl)uronium hexafluorophosphate (HBTU) were obtained from Peptides International Inc (Kentucky). Aminomethyl-resin was prepared from Biobeads S-X1 (BioRad, California) by published methods.[1] Side-chain protecting groups for Boc-amino acids used were Arg(Tos), Asn(Xan), Asp(OcHex), Cys(4-Me-Bzl), Glu(OcHex), His(DNP), Lys(2Cl-Z), Ser(Bzl), Thr(Bzl), Tyr(2Br-Z). Side-chain protecting groups for Fmoc-amino acids used were: Arg(Pbf), Asn(Trt), Ser(tBu). Boc-L-1,3-thiazolidine-4-carboxylic acid (Boc-Thz) was obtained from Bachem. Boc-L-His(DNP) was purchased from Chem-Impex international Inc. N-(2-Chlorobenzyloxycarbonyloxy)succinimide (Z(2-Cl)-OSu) was obtained from Chem-Impex International Inc (Wood Dale, IL). Trifluoroacetic acid (TFA) was purchased from Halocarbon products (New Jersey). N,N-Diisopropylethylamine (DIEA) was obtained from Applied Biosystems. Hydrogen fluoride (HF) was purchased from Matheson. Piperidine was purchased from Sigma-Aldrich. N,N-dimethylformamide (DMF) was obtained from Pharmco-AAper (ACS grade). Diethyl ether (ACS certified), dichloromethane (DCM, HPLC grade), acetonitrile (HPLC-grade), and guanidine hydrochloride were purchased from Fisher. All other reagents were purchased from Sigma-Aldrich and were of the purest grade available.

2. Analytical LC-MS

Analytical reversed phase HPLC-MS was performed using an Agilent 1100 series HPLC system equipped with an online MSD ion trap. Conditions for the chromatographic separations are provided under each chromatogram. Masses were obtained by online electrospray mass spectrometry. All MS data shown were collected across the entire principal UV absorbing peak in each chromatogram.

3. Preparative Reverse-phase HPLC purifications

Crude peptides and ligation reaction products were either dissolved in 6M GuHCl or in suitable solvent mixtures (maximum of 10% B), acidified to pH 2-3, and filtered (0.22μ). The clear solution was then loaded onto either a C4 (10x100 mm) in-house packed column or a C18 (9.4x250 mm) Zorbax column, and the peptide components eluted with flow rate of 5 mL/min using a shallow gradient of solvent B in solvent A (solvent A = 0.1% TFA in water, solvent B = 0.08% TFA in acetonitrile). Fractions containing the desired purified peptides were identified by analytical LC and mass spectrometry, then combined and lyophilized.

Ant insulin ILP-2 Sequence (A) and proposed ester linked precursor molecule (B)

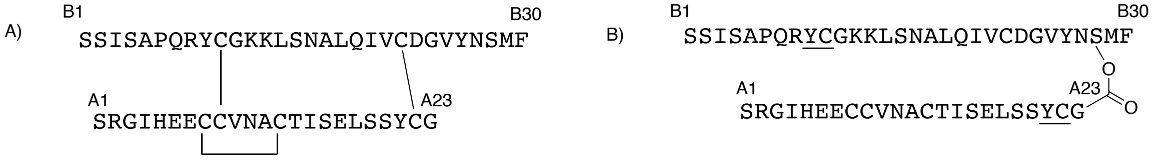

Three peptide segments chosen for the synthesis based on the disconnections at underlined residues (above) were:

Ser^{B1}-Tyr^{B9}-$^{\alpha}COSR$ (**1**), Ser^{A1}-Tyr^{A21}-$^{\alpha}COSR$ (**2**), and Cys(Thz)^{B10}-Ser^{B28}[O(Gly^{A23}-Cys^{A22})]-Phe^{B30} (**3**)

4. Synthesis of Fmoc-Ser[O(Boc-Gly)]-OH dipeptide:

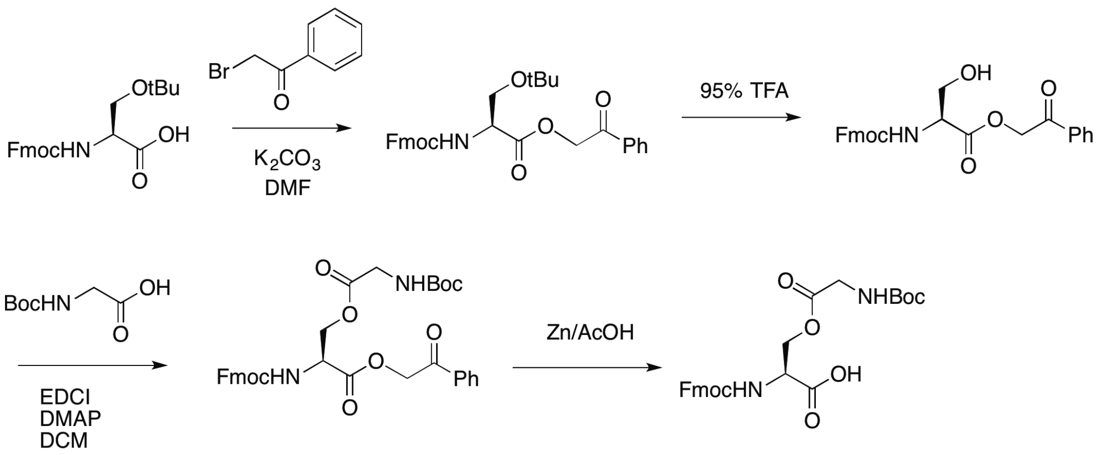

Fmoc-Ser(OtBu)-OPac

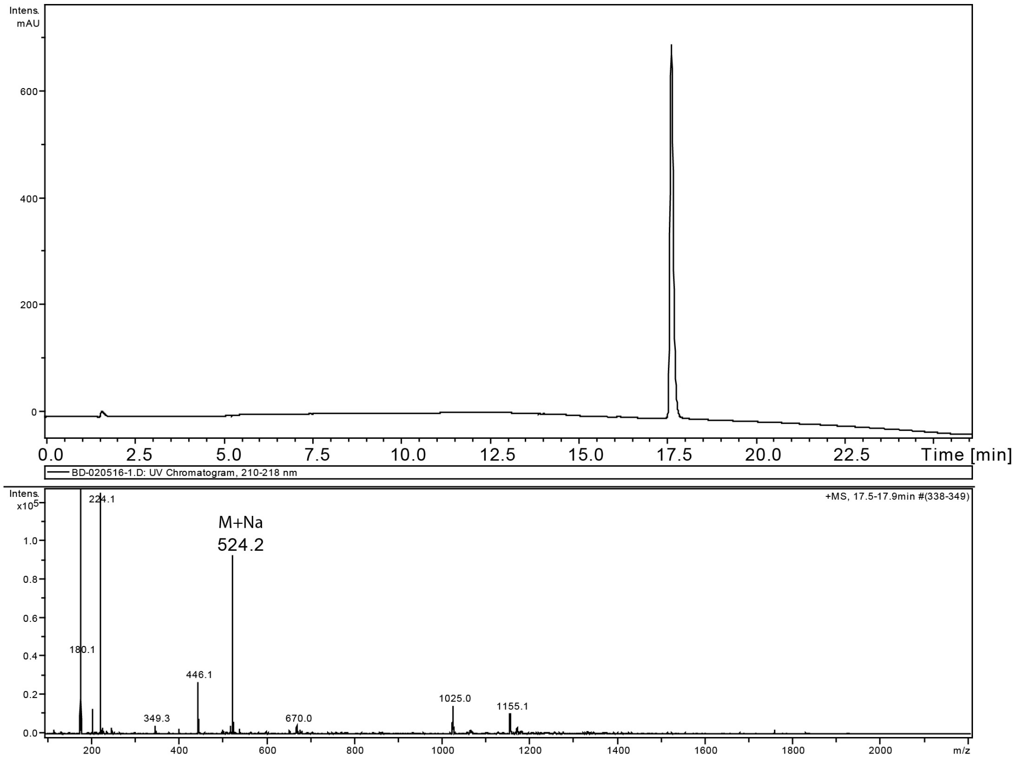

Figure S1. Analytical LC-MS data of Fmoc-Ser(OtBu)-OPac. (Top panel) Reverse phase HPLC separations were performed on a C4 (4.6x150 mm) column at 40 °C, using a linear gradient (5−45%) of solvent B in solvent A over 20 min (solvent A = 0.1% TFA in water, solvent B = 0.08% TFA in acetonitrile) at a flow rate of 1.0 mL/min with detection by UV absorption at 214 nm. (Bottom panel) Online ESI-MS spectra, taken across the whole of the main UV peak.

Fmoc-Ser(OH)-OPac

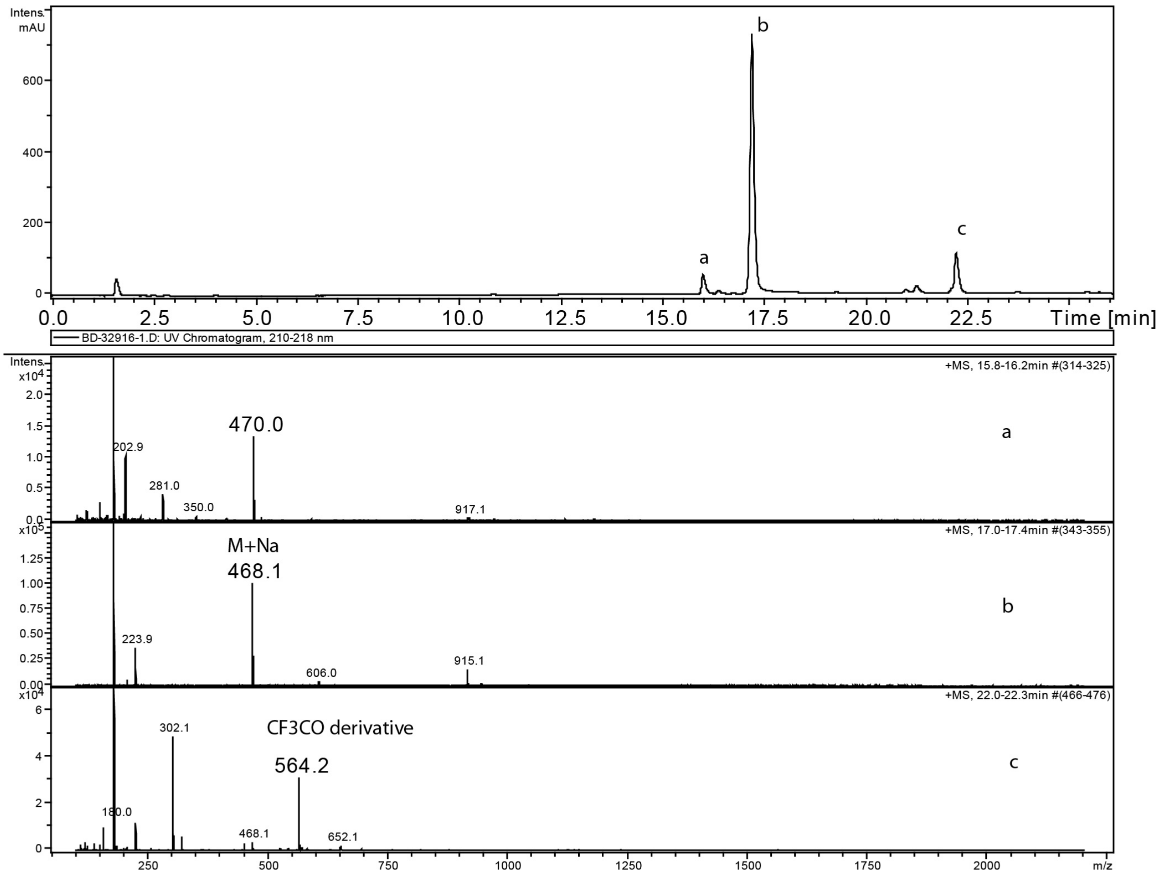

Figure S2. Analytical LC-MS data for Fmoc-Ser(OH)-OPac. (Top panel) Reverse phase HPLC separations were performed on a C4 (4.6x150 mm) column at 40 °C, using a linear gradient (5−45%) of solvent B in solvent A over 20 min (solvent A = 0.1% TFA in water, solvent B = 0.08% TFA in acetonitrile) at a flow rate of 1.0 mL/min with detection by UV absorption at 214 nm. (Bottom panel) Online ESI-MS spectra, taken across the whole of each of the UV peaks a-c.

Fmoc-Ser[O(Boc-Gly)]-OPac

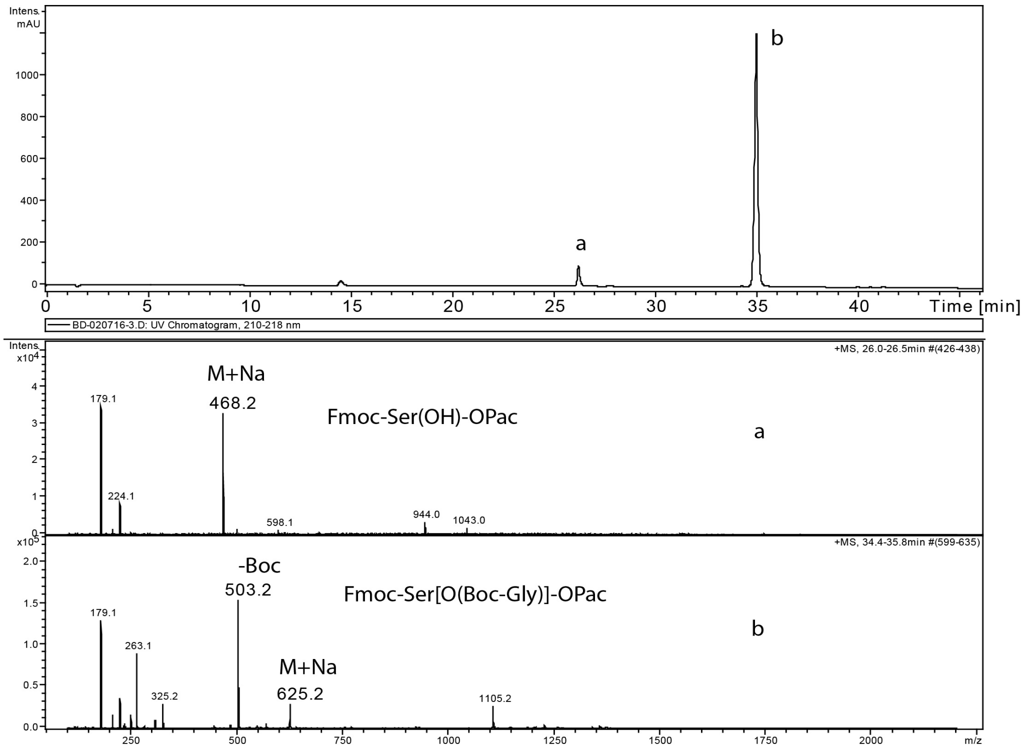

Figure S3. Analytical LC-MS data for Fmoc-Ser[O(Boc-Gly)]-OPac. (Top panel) Reverse phase HPLC separations were performed on a C4 (4.6x150 mm) column at 40 °C, using a linear gradient (5−45%) of solvent B in solvent A over 40 min (solvent A = 0.1% TFA in water, solvent B = 0.08% TFA in acetonitrile) at a flow rate of 1.0 mL/min with detection by UV absorption at 214 nm. (Bottom panel) Online ESI-MS spectra, taken across the whole of each of the UV peaks a-b.

Fmoc-Ser[O(Boc-Gly)]-OH

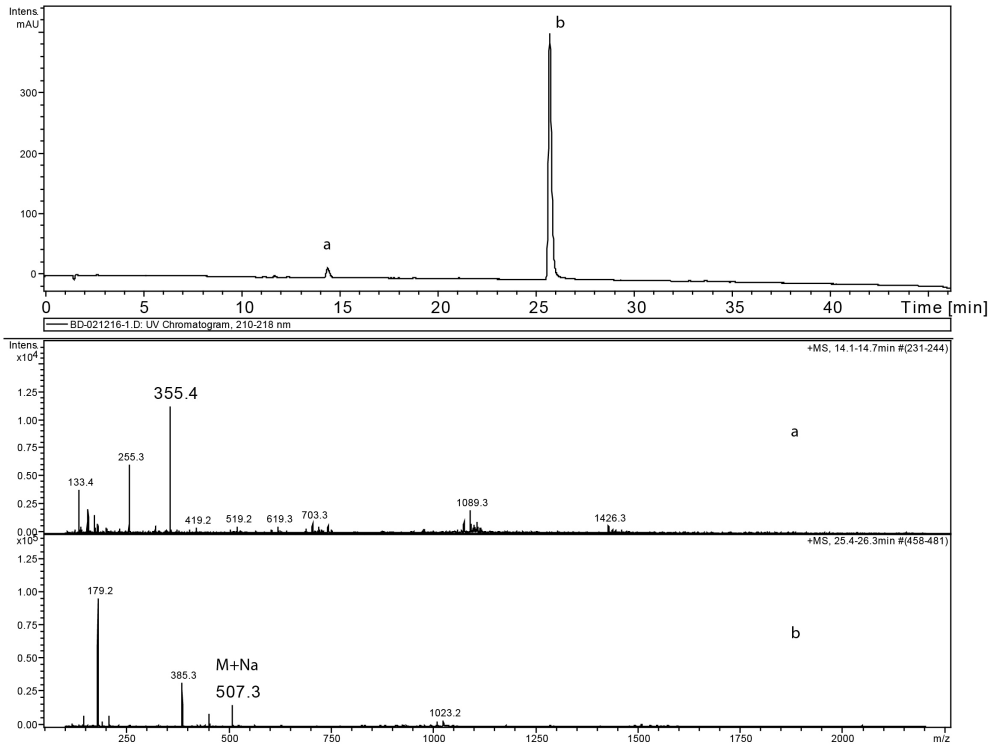

Figure S4. Analytical LC-MS data for Fmoc-Ser[O(Boc-Gly)]-OH. (Top panel) Reverse phase HPLC separations were performed on a C4 (4.6x150 mm) column at 40 °C, using a linear gradient (5−45%) of solvent B in solvent A over 40 min (solvent A = 0.1% TFA in water, solvent B = 0.08% TFA in acetonitrile) at a flow rate of 1.0 mL/min with detection by UV absorption at 214 nm. (Bottom panel) Online ESI-MS spectra, taken across the whole of each of the UV peaks a-b.

5. Synthesis of peptide segments 1-3

Synthesis of Ser^{B1}-Tyr^{B9}-SCH_2CO-Ala (1)

After assembly of the target sequence, the final N^{α}-Boc group was removed and the peptide was cleaved from resin with simultaneous removal of all the protecting groups with the exception of His(DNP), using 5% p-cresol/HF at 0 °C for 1 h. After careful evaporation of HF at 0 °C, the resulting residue was treated with ice-cold ether. After preparative HPLC as described in METHODS, purified peptide was obtained in 22.5% yield (52 mg, 45.1 μmol). LCMS (ESI) data: Obsd. 1153.2 ± 0.2 Da; calculated (average isotopes), 1153.2 Da.

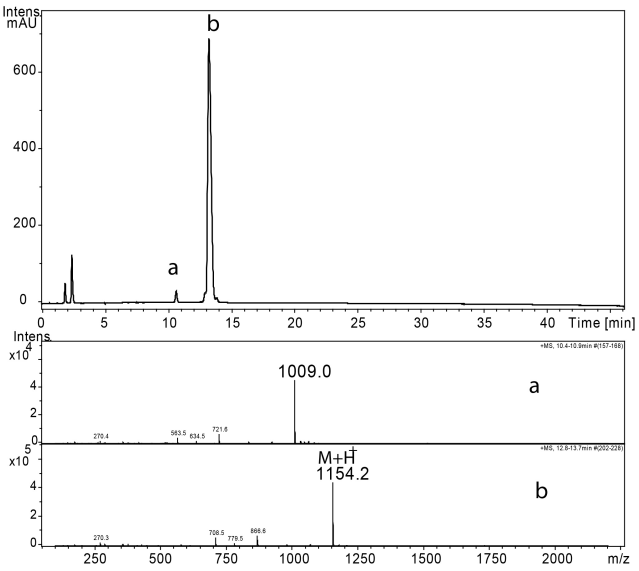

Figure S5. Analytical LC-MS data for Ser^{B1}-Tyr^{B9}-SCH_2CO-Ala. (Top panel) Reverse phase HPLC – The chromatographic separations were performed on a C4 (4.6x150 mm) column at 40 °C, using a linear gradient (5−45%) of solvent B in solvent A over 40 min (solvent A = 0.1% TFA in water, solvent B = 0.08% TFA in acetonitrile) at a flow rate of 1.0 mL/min with detection by UV absorption at 214 nm. (Bottom panel) Online ESI-MS spectra, taken across the whole of each of the UV peaks. Peak a: deletion of Ala and $-SCH_2CO$ moieties (-145 Da)].

Synthesis of Ser^{A1}-Tyr^{A21}-SCH_2CO-Arg_4-Ala (2)

Owing to high hydrophobicity and difficulty in handling and purification of Ser^{A1}-Tyr^{A21}-SCH_2CO-Ala thioester, an Arg_4-tag was incorporated in the thioester-leaving group in an optimized synthesis.[2] Boc chemistry SPPS ‘in situ neutralization’ protocols as described above were carried out on Boc-Ala-OCH_2-Pam-resin (0.2 mmol)). After

assembly of the target sequence by SPPS, the final N$^{\alpha}$-Boc group was removed and the peptide was cleaved from resin with simultaneous removal of all the protecting groups with the exception of His(DNP), using 5% p-cresol/HF at 0 °C for 1 h. After careful evaporation of HF at 0 °C, the resulting residue was treated with ice-cold ether. After preparative HPLC as described in METHODS, purified peptide was obtained in 11% yield (72 mg, 22.2 μmol). LCMS (ESI) data: Obsd. 3237.0 ± 0.3 Da; calculated (average isotopes), 3237.5 Da.

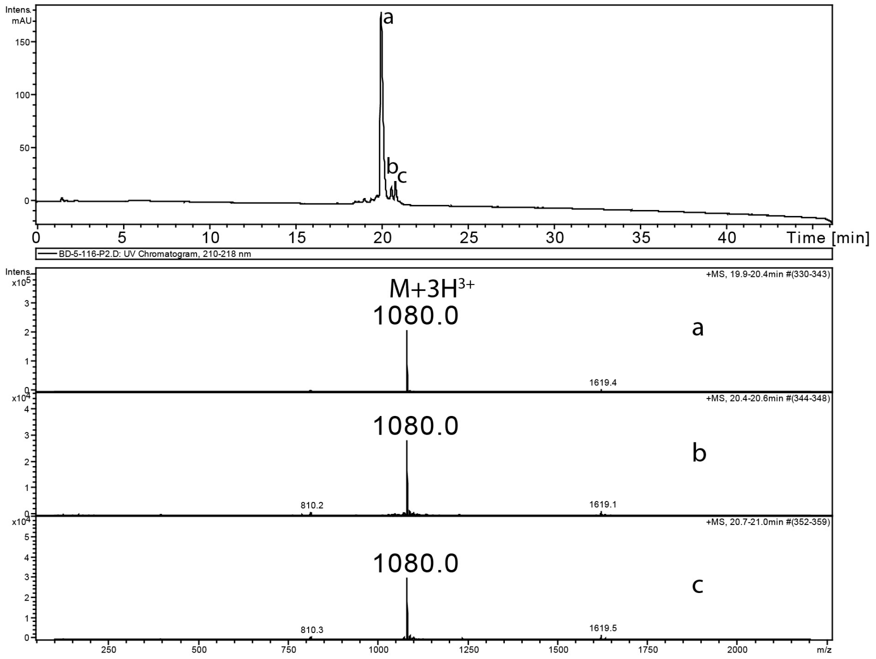

Figure S6. Analytical LC-MS data for purified Ser^{A1}-Tyr^{A21}-SCH$_2$CO-Arg$_4$-Ala. (Top panel) Reverse phase HPLC separations were performed on a C4 (4.6x150 mm) column at 40 °C, using a linear gradient (5−45%) of solvent B in solvent A over 40 min (solvent A = 0.1% TFA in water, solvent B = 0.08% TFA in acetonitrile) at a flow rate of 1.0 mL/min with detection by UV absorption at 214 nm. (Bottom panel) Online ESI-MS spectra, taken across the whole of each of the UV peaks.

Synthesis of Thz^{B10}-Ser^{B28}[O(Gly^{A23}-Cys^{A22})]-Phe^{B30} (3)

After Boc-Met coupling to deprotected Boc-Phe-OCH$_2$-Pam resin (0.5 mmol), the dipeptide Fmoc-Ser[O(Boc-Gly)]-OH was coupled using the following conditions: Fmoc-Ser[O(Boc-Gly)]-OH (850 mg, 1.75 mmol), 1.6 mmol HATU (608 mg), 2.6 mmol DIEA (450 μL) in DMF (3.5 mL) for 1h. Boc-Cys(4MeBzl) was coupled after removal of the Boc-group from N$^{\alpha}$-Gly^{A23} by TFA treatment. The Boc group of N$^{\alpha}$-Cys^{A22} was then

removed and reprotected as Z(2-Cl). Conditions used were: Z(2-Cl)-OSu (1.42 g, 5.0 mmol), DIEA (5.5 mmol, 962 µL) and DMF (3.5 mL).

Fmoc-deprotection at the Ser^{B28} was carried out by 20% piperidine/DMF treatment. Following removal of the SerB28 Fmoc group, Boc SPPS cycles were carried out from Asn^{B27} through Thz^{B10} using the Boc chemistry 'in situ neutralization' protocols described above. After assembly of the target sequence by SPPS, the final N^{α}-Boc group was removed and the peptide was cleaved from resin with simultaneous removal of all the protecting groups using 5% p-cresol/HF at 0 °C for 1 h. After preparative HPLC as described in METHODS, purified target peptide was obtained in 5.1% yield (63 mg, 25.6 µmol). *Upon removal of the N-alpha Fmoc of SerB28, an internal nucleophilic attack by the SerB28 alpha-amine on the ester moiety, favored by a five-membered ring geometry, would lead to the terminated peptide CysA22-GlyA23-SerB28-MetB29-PheB30 following the final global deprotection and cleavage. We did not observe such a byproduct, but on the other hand we were not explicitly looking for it and the suggested side reaction may have contributed to the low overall yield of the purified branched depsipeptide segment.*

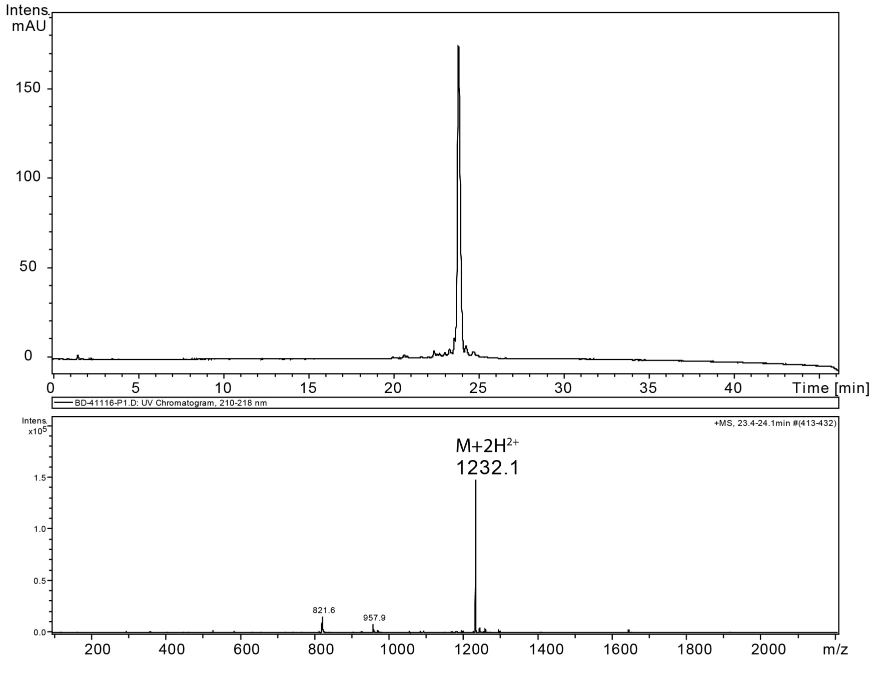

Figure S7. Analytical LC-MS data for purified Thz^{B10}-Ser^{B28}[O(Gly^{A23}-Cys^{A22})]-Phe^{B30}. (Top panel) Reverse phase HPLC separations were performed on a C4 (4.6x150 mm) column at 40 ^{o}C, using a linear gradient (5−45%) of solvent B in solvent A over 40 min (solvent A = 0.1% TFA in water, solvent B = 0.08% TFA in acetonitrile) at a flow rate of 1.0 mL/min with detection by UV absorption at 214 nm. (Bottom panel) Online ESI-MS spectra, taken across the whole of main UV peak. LCMS (ESI) data: Obsd. 2462.2 ± 0.3 Da; calculated (average isotopes), 2462.9 Da.

6. Synthesis of ant ILP2 full-length depsipeptide 5

Initially, peptide segments Ser^{A1}-Tyr^{A21}-SCH$_2$CO-Arg$_4$-Ala (**2**, 2.0 mg, 0.618 μmol, note: with His-DNP protecting group) and Thz^{B10}-Ser^{B28}[O(Gly^{A23}-Cys^{A22})]-Phe^{B30} (**3**, 1.45 mg, 0.588 μmol) were reacted under 100 mM 4-mercaptophenylacetic acid (MPAA), pH 7.0 (6M GuHCl, 0.1M NaH$_2$PO$_4$(Pi)) conditions. An aliquot was analyzed after 4h by treating with 50 mM Tris(2-carboxyethyl)phosphine hydrochloride (TCEP.HCl) in 6M GuHCl, 0.1M Pi. (Note: The His 2,4-dinitrophenyl group was spontaneously removed under the ligation conditions). 50 mM TCEP was added and the pH adjusted to 7.0 and allowed to stand for 30 min to reduce the disulfide by-products formed during the ligation reaction. Then in the same pot, methoxylamine hydrochloride (MeONH$_2$.HCl) (200 mM, 3.34 mg) was added and pH adjusted to 4.0 in order to effect the Thz– to Cys– conversion. Thz to Cys conversion was complete after 12 h as monitored by LCMS and was indicated by mass difference of 12.0 Da. To this reaction was then added Ser^{B1}-Tyr^{B9}-SCH$_2$CO-Ala (**1**, 0.68 mg, 0.588 mmol), 100 mM MPAA (3.36 mg) and 50 mM TCEP.HCl (2.86 mg) and the pH adjusted to 7.0. The ligation reaction was allowed to proceed for 20 hours under these conditions. No desired product **5** remained; products due to the cleavage reactions at Asn-Ser (**5a**, **5b** and **6b**) were formed. Analytical LCMS data for this series of one-pot reactions are presented below (**Figure S8**).

A)

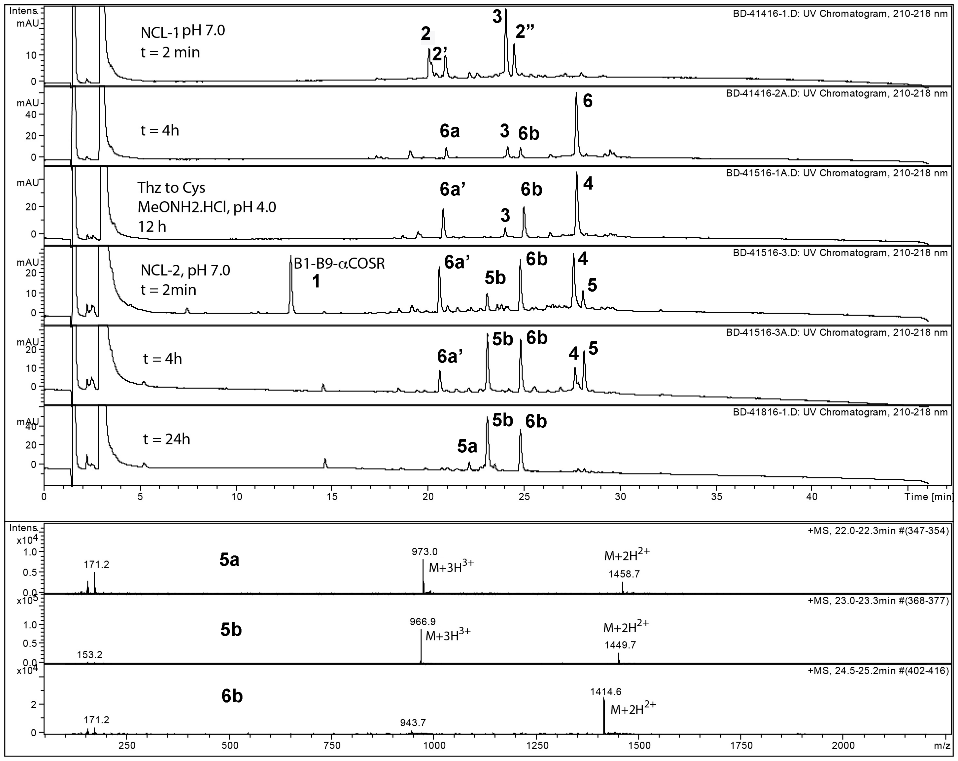

B)

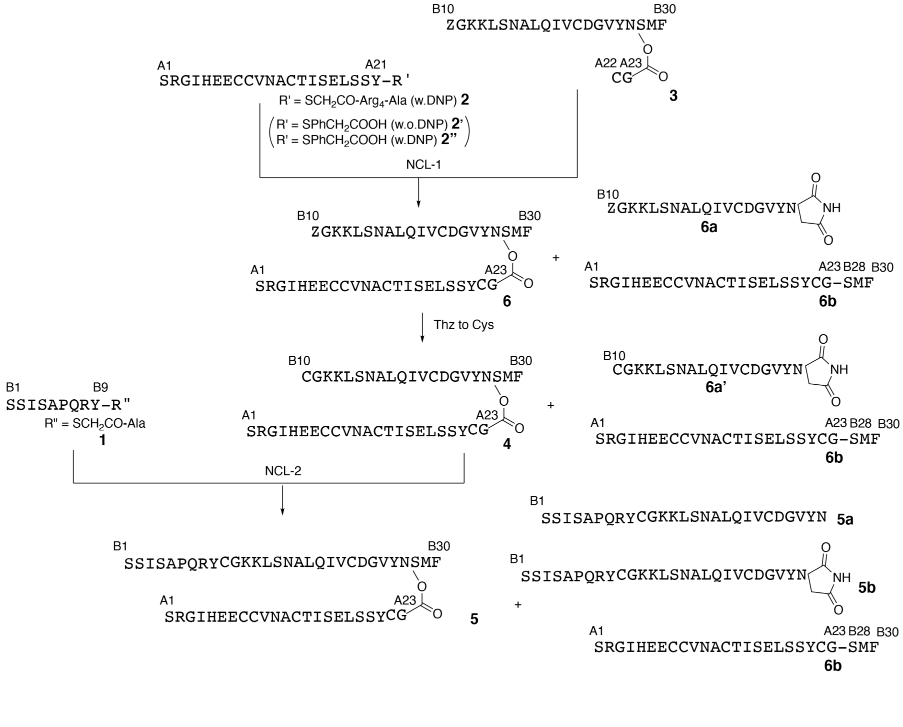

Figure S8. A) Analytical LC-MS data for Synthesis of Ant insulin full-length peptide. (Top panel) Reverse phase HPLC – The chromatographic separations were performed on a C4 (4.6x150 mm) column at 40 °C, using a linear gradient (5−45%) of solvent B in solvent A over 40 min (solvent A = 0.1% TFA in water, solvent B = 0.08% TFA in acetonitrile) at a flow rate of 1.0 mL/min with detection by UV absorption at 214 nm. (Bottom panel) Online ESI-MS spectra, taken across the whole of each of the UV peaks. B) Schematic structures of the observed products.

While these initial reaction conditions failed to produce any full-length material, the modified procedures described in main text METHODS significantly suppressed the cleavage reactions **(Figure S9)**. Analysis of peak ratios between the peak at 24.8 min (compound **6b**) and peaks at 27-28 min (compound **4**, **5** or **6**) clearly shows significant cleavages even under optimized reaction conditions (**Table S1**).

Table S1. Time course of cleavage products during one-pot native chemical ligation steps.

		% peak (by area)	
	Time of reaction	Cleavage fragment (24.8 min)	Desired products (~27-28 min)
NCL-1	2h	13.6	86.4
pH 7.0	2.5 h	18.1	81.9
1) 50 mM TCEP.HCl	1h	26.6	73.4
pH 7.0, 5 min	2h	26.5	73.5
2) Thz to Cys	3.5h	27.1	72.9
pH 4.0	5h	27.3	72.7
NCL-2	2min	31.3	68.7
pH 6.7	1h	47.7	52.3
	1.5h	46.2	53.8

A)

B)

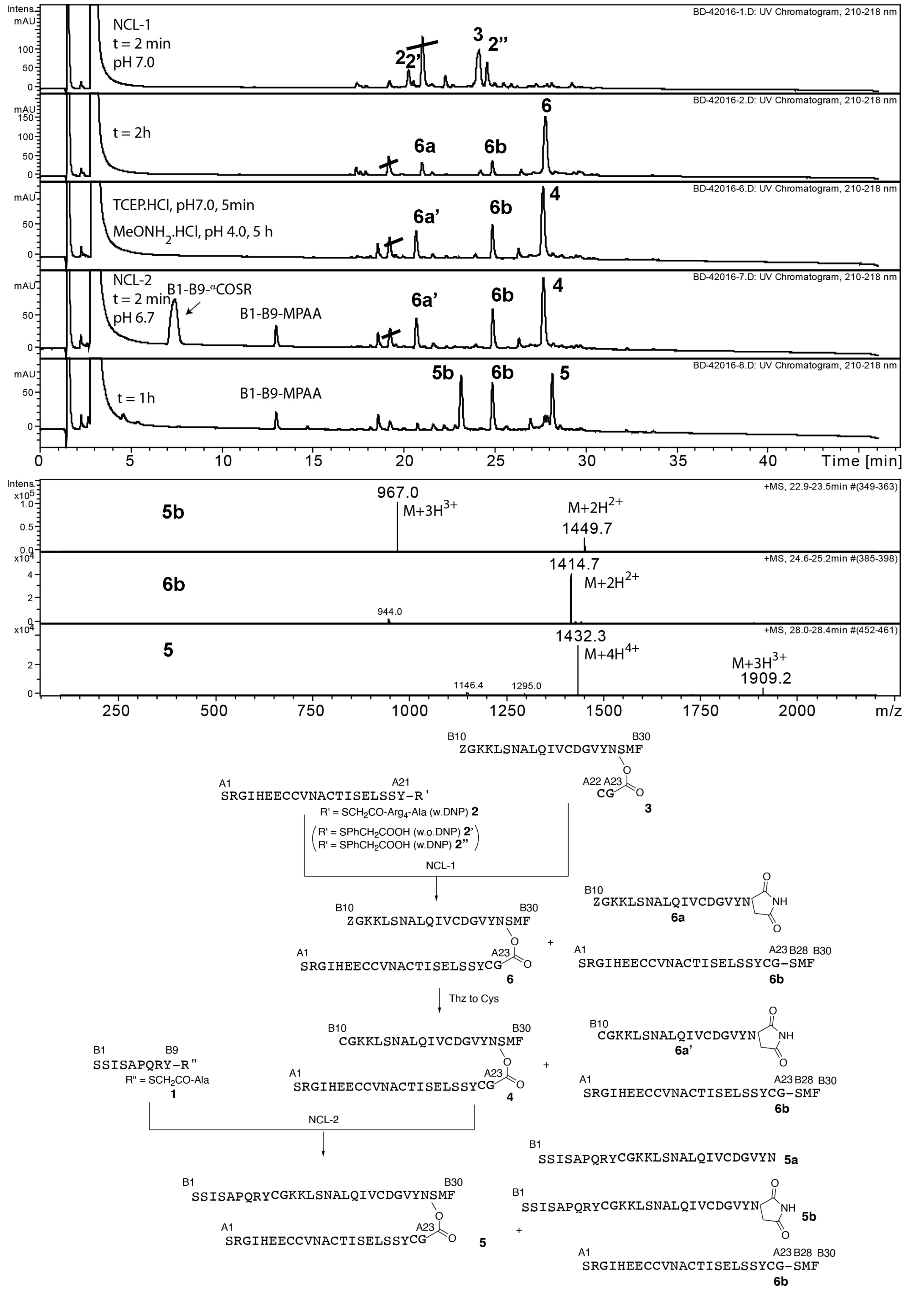

Figure S9. A) Analytical LC-MS data for Synthesis of Ant insulin full-length peptide <u>using modified conditions</u>. (Top panel) Reverse phase HPLC separations were performed on a C4 (4.6x150 mm) column at 40 °C, using a linear gradient (5−45%) of solvent B in solvent A over 40 min (solvent A = 0.1% TFA in water, solvent B = 0.08% TFA in acetonitrile) at a flow rate of 1.0 mL/min with detection by UV absorption at 214 nm. (Bottom panel) Online ESI-MS spectra, taken across the whole of each of the UV peaks. B) Schematic structures of the observed products.

The crude material was subjected to preparative HPLC using a fast gradient on a C4(10x100 mm) column to give partially purified full-length depsipeptide (d, 28.2 min) (**Figure 10**)

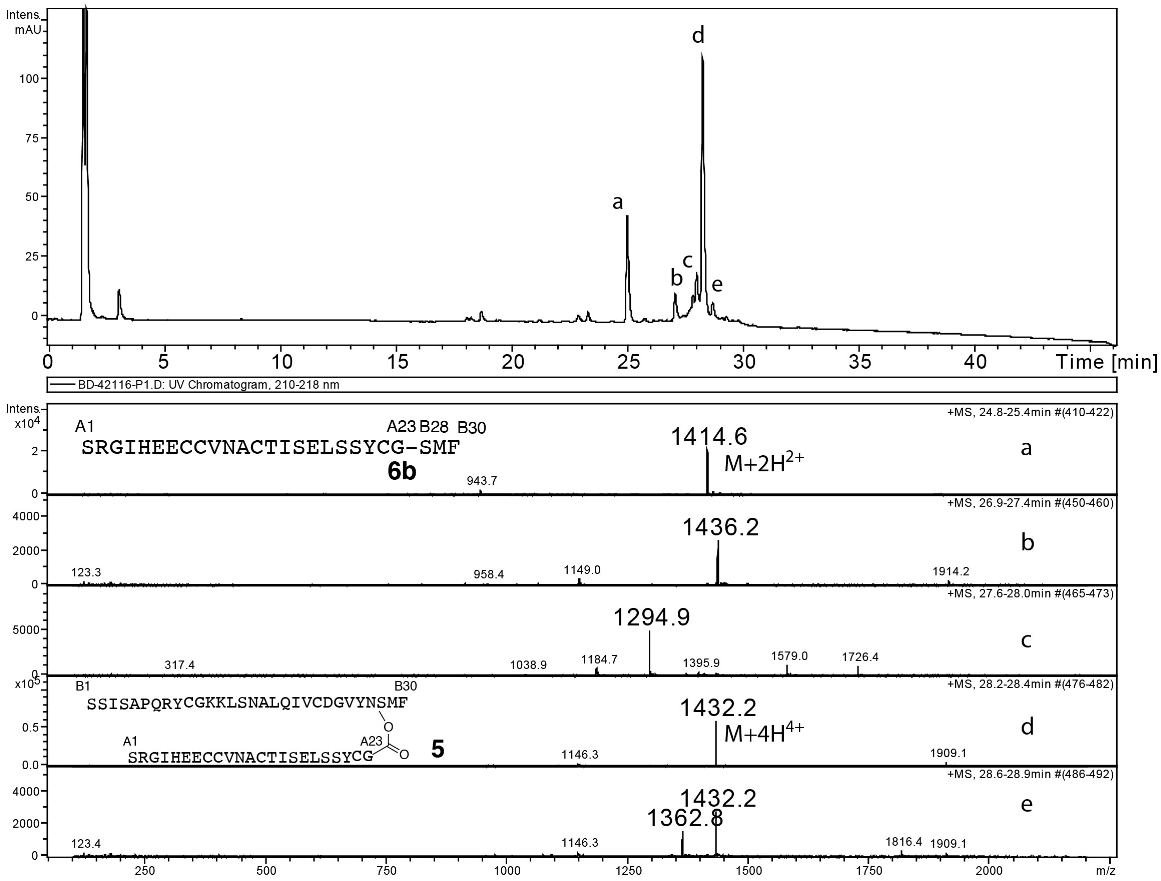

Figure S10. Analytical LC-MS data for Purified Ant insulin full length polypeptide. (Top panel) Reverse phase HPLC separations were performed on a C4 (4.6x150 mm) column at 40 °C, using a linear gradient (5−45%) of solvent B in solvent A over 40 min (solvent A = 0.1% TFA in water, solvent B = 0.08% TFA in acetonitrile) at a flow rate of 1.0 mL/min with detection by UV absorption at 214 nm. (Bottom panel) Online ESI-MS spectra, taken across the whole of each of the UV peaks.

7. Stability of ester bond under LiOH saponification conditions

Support for the rearrangement of the once-cleaved ester-containing fragment was evident when the peptide Thz^{B10}-Ser^{B28}[O(Gly^{A23}-Cys^{A22})]-Phe^{B30} (**3**) was treated under 25 mM LiOH (pH ~12) saponification conditions in water at 4 °C. The cleavage reaction was rapid in this case. Two main products Cys^{A22}-Gly^{A23}-Ser^{B28}-Met^{B29}-Phe^{B30} and Thz^{B10}-Asn^{B27}-OH were formed presumably due to the base catalyzed cleavage at the -Asn^{B27}-Ser^{B28}[O(Gly^{A23}-Cys^{A22})] bond, followed by two other reactions: rapid intramolecular nucleophilic attack by the Ser^{B28} alpha amino group on the ester bond carbonyl of Gly^{A23}, to give the amide linked product, stable to hydrolysis; and, rapid base catalyzed hydrolysis of the C-terminal B27 aspartimide, to give the Thz^{B10}-Asn^{B27}-OH. Such a series of reactions would explain why the ester bond initially believed to be present in the Cys^{A22}-Gly^{A23}-Ser^{B28}-Met^{B29}-Phe^{B30} cleavage fragment did not hydrolyze under these extremely basic conditions.

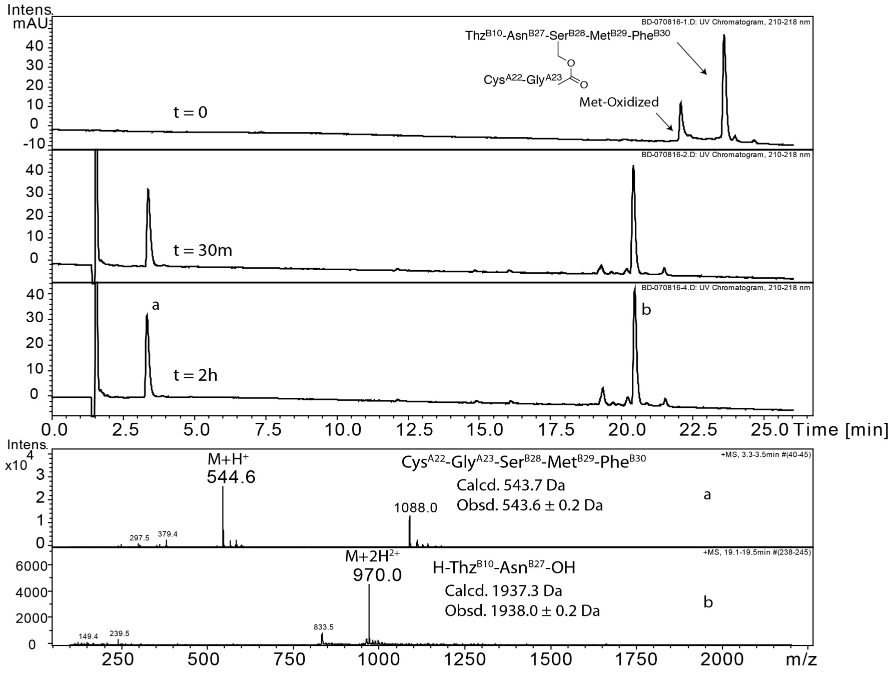

Figure S11. Analytical LC-MS data for LiOH saponification of Thz^{B10}-Ser^{B28}[O(Gly^{A23}-Cys^{A22})]-Phe^{B30} (**3**) at 4° C. (Top panel) Reverse phase HPLC separations were performed on a C4 (4.6x150 mm) column at 40 °C, using a linear gradient (5−28%) of solvent B in solvent A over 23 min (solvent A = 0.1% TFA in water, solvent B = 0.08% TFA in acetonitrile) at a flow rate of 1.0 mL/min with detection by UV absorption at 214 nm. (Bottom panel) Online ESI-MS spectra, taken across the whole of each of the UV peaks.

8. Folding of ant ILP2 full length depsipeptide

Folding of the crude full-length depsipeptide was carried out in an analytical scale under the following conditions, which were based on the conditions used to folded human ester insulin:[3] 1.5 M GuHCl, 20 mM Tris, 8 mM Cysteine, 1 mM Cystine, pH 7.6 at 4 ºC (**Figure S12**) and room temperature (**Figure S13**). In both of the conditions folding was complete within 1 h, however better results were observed in 4 ºC. Cleavage at the Asn^{B27}-Ser^{B28} site was also seen under the folding conditions.

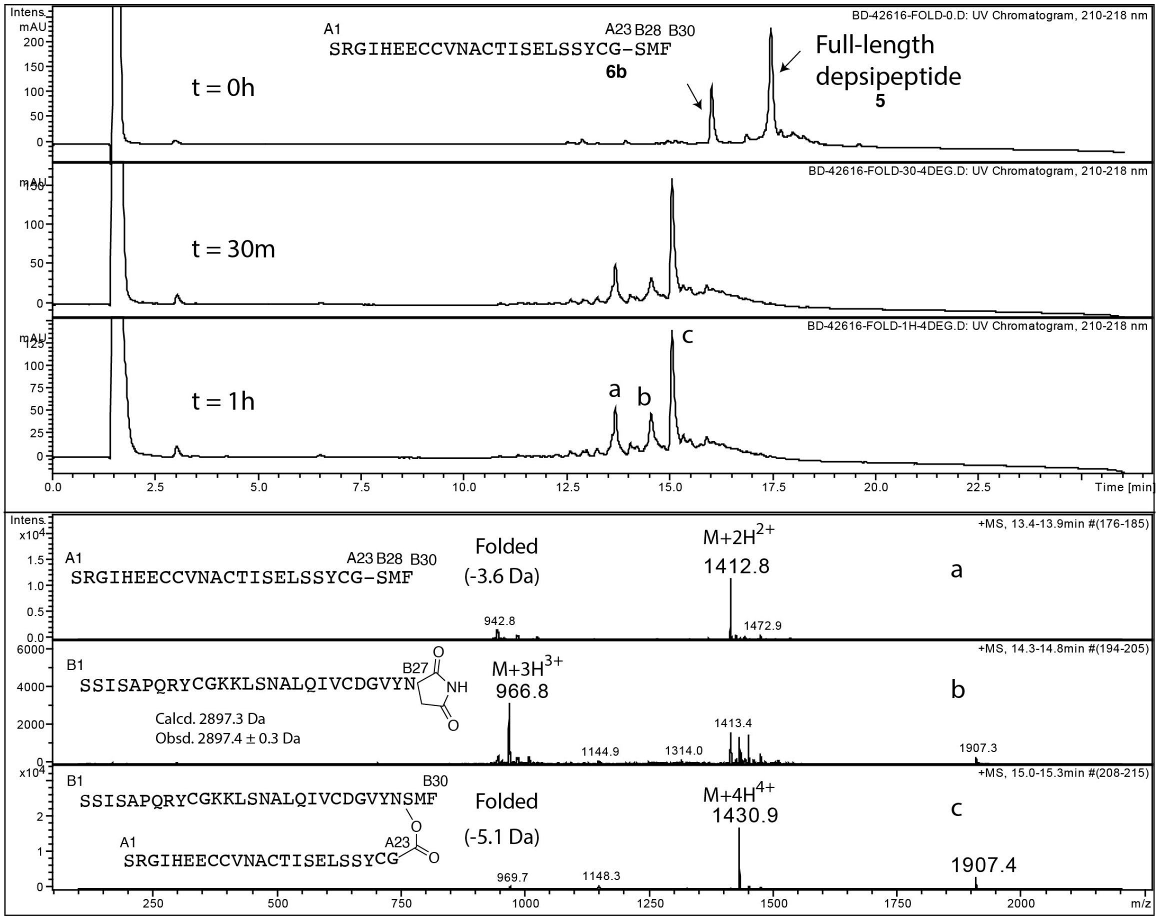

Figure S12. Analytical LC-MS data for folding of Ant insulin full length polypeptide ***at 4 ºC***. (Top panel) Reverse phase HPLC separations were performed on a C4 (4.6x150 mm) column at 40 ºC, using a linear gradient (5−45%) of solvent B in solvent A over 20 min (solvent A = 0.1% TFA in water, solvent B = 0.08% TFA in acetonitrile) at a flow rate of 1.0 mL/min with detection by UV absorption at 214 nm. (Bottom panel) Online ESI-MS spectra, taken across the whole of each of the UV peaks.

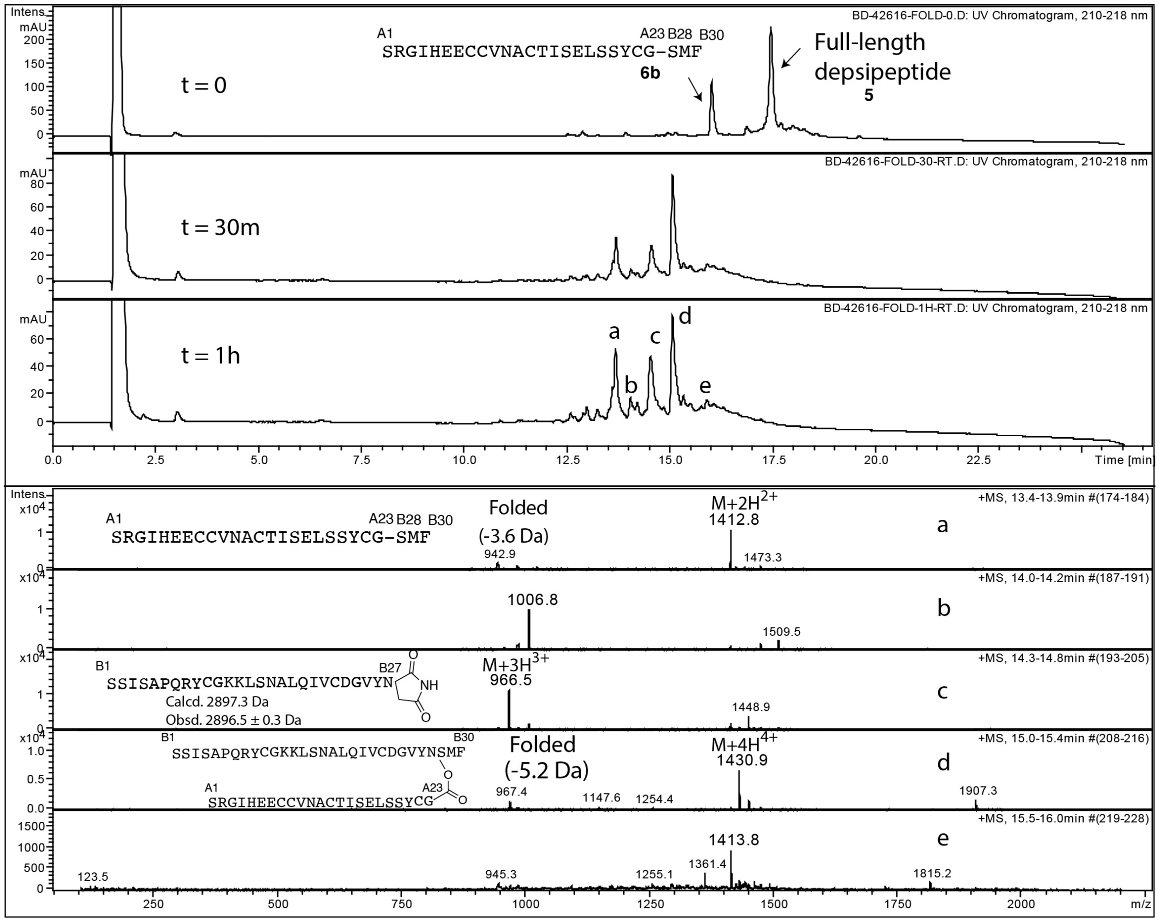

Figure S13. Analytical LC-MS data for folding of Ant insulin full length polypeptide ***at room temperature***. (Top panel) Reverse phase HPLC separations were performed on a C4 (4.6x150 mm) column at 40 °C, using a linear gradient (5−45%) of solvent B in solvent A over 20 min (solvent A = 0.1% TFA in water, solvent B = 0.08% TFA in acetonitrile) at a flow rate of 1.0 mL/min with detection by UV absorption at 214 nm. (Bottom panel) Online ESI-MS spectra, taken across the whole of each of the UV peaks.

9. Synthesis of Ac-Phe-Arg-Ala-Asn-Ser(OAc)-Phe-Arg-Ala

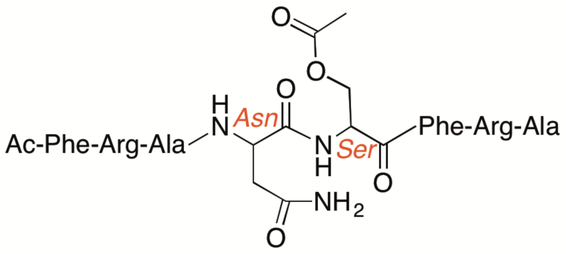

Ac-FRANS(OAc)FRA-OH

The Fmoc chemistry stepwise SPPS protocol used was: scale 0.1 millimol H-Ala-O-2-Chlorotrityl-(S-DVB)resin; DMF washes: 10 sec flow, 1x1min batch; Fmoc-AA (0.55 mmol) dissolved in 0.5M HBTU in DMF (1 mL, 0.5 mmol); 0.75 mmol DIEA (131 μL) was added; after 30 sec activation, the solution was added to the peptide-resin; coupling 30 min. N^{α}Fmoc removal: 20% v/v piperidine/DMF 2x5min batch treatments.

After addition of Fmoc-Arg and Fmoc-Phe residues to the H-Ala-O-2-Chlorotrityl-(S-DVB)resin, the Fmoc group was removed and side chain unprotected Fmoc-Ser was coupled. Before Fmoc-deprotection at the N^{α}-Ser, the free side chain hydroxyl group of serine was acetylated using 20% v/v Ac_2O/pridine for 1h. Then the remainder of the sequence from Fmoc-Asn through Fmoc-Phe was assembled by Fmoc chemistry stepwise SPPS using the HBTU/DIEA mediated coupling protocol described above. After final N^{α}Fmoc deprotection at the N-terminus, the free alpha-amino group was acetylated using 20% Ac_2O/pyridine for 30 min. The peptide-resin was then washed with DMF followed by DCM.

The product peptide was cleaved from the -Ala-2-Chlorotrityl-(S-DVB)resin and simultaneously deprotected by subjecting it to TFA/TIPS/water/EDT (95:2:2:1 v/v) conditions at ambient temperature. After 3h, the cleavage mixture was collected in a RB flask and the resin was washed once again with 1mL TFA. The filtrate was evaporated and the solid residue was triturated with 1:1 ice-cold ether and hexane (2x). The resultant white precipitate was then dissolved directly in 6M GuHCl and purified using RP prep-HPLC on C18 column (9.4x250 mm) to give the target peptide in 14.4% yield (15.2 mg, 14.4 μmol).

Mass calculated for Ac-Phe-Arg-Ala-Asn-Ser(OAc)-Phe-Arg-Ala, 1052.2 Da (average isotope composition), Obsd. 1052.1 ± 0.1 Da.

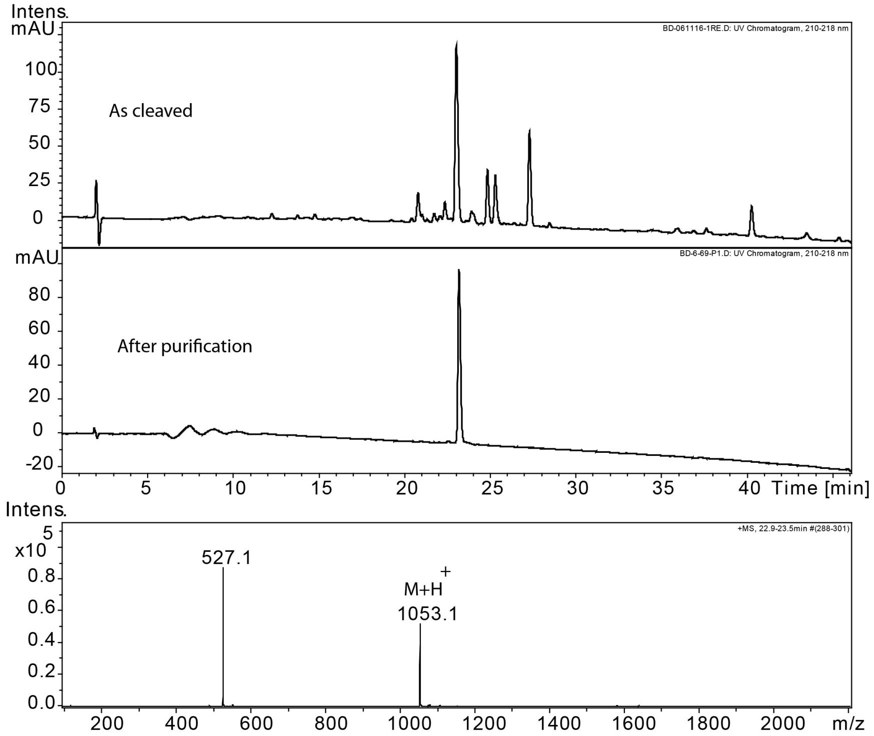

Figure S14. LC-MS data for the synthesis of Ac-Phe-Arg-Ala-Asn-Ser(OAc)-Phe-Arg-Ala. (Top panel) Reverse phase HPLC chromatographic separations were performed on a C8 (4.6x150 mm) column at 40 °C, using a linear gradient (1−41%) of solvent B in solvent A over 40 min (solvent A = 0.1% TFA in water, solvent B = 0.08% TFA in acetonitrile) at a flow rate of 1.0 mL/min with detection by UV absorption at 214 nm. (Bottom panel) Online ESI-MS spectrum taken across the whole of the main UV peak.

10. Stability of Ac-Phe-Arg-Ala-Asn-Ser(OAc)-Phe-Arg-Ala

Ac-Phe-Arg-Ala-Asn-Ser(OAc)-Phe-Arg-Ala (1.05 mg, 4 mM) was dissolved in 250 µL of 6 M GuHCl, 0.1 M Pi buffer at pH 7.0, and treated with 250 µL of 100 mM TCEP.HCl in 6M GuHCl, 0.1 M Pi buffer (pH 7.0) and an aliquot was analyzed by LC-MS after acidifying to pH 3.0 at various time intervals.

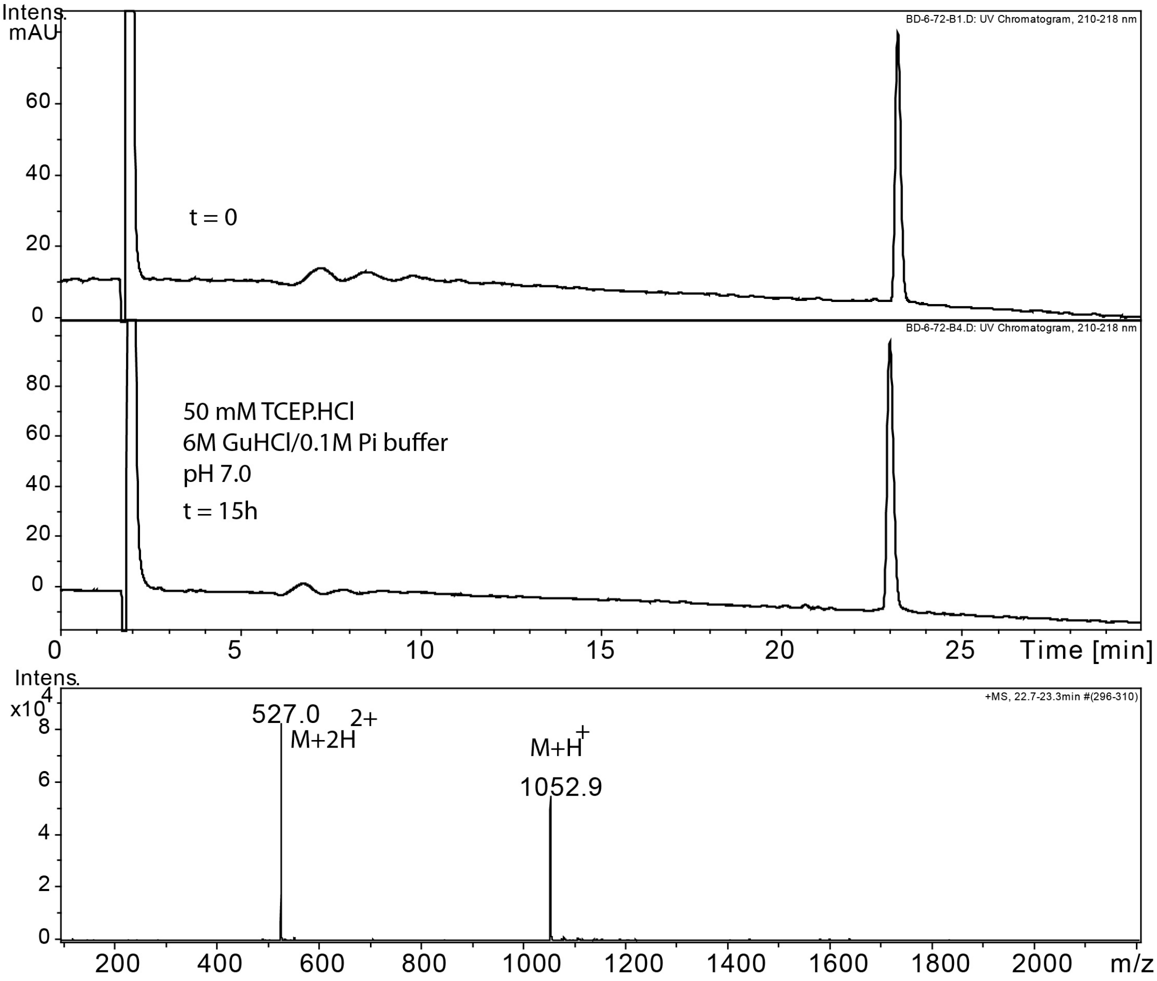

Figure S15. LC-MS data for the stability studies of Ac-Phe-Arg-Ala-Asn-Ser(OAc)-Phe-Arg-Ala. (Top panel) Reverse phase HPLC chromatographic separations were performed on a C8 (4.6x150 mm) column at 40 ºC, using a linear gradient (1−28%) of solvent B in solvent A over 27 min (solvent A = 0.1% TFA in water, solvent B = 0.08% TFA in acetonitrile) at a flow rate of 1.0 mL/min with detection by UV absorption at 214 nm. (Bottom panel) Online ESI-MS spectrum taken across the whole of the main UV peak.

11. Synthesis of Ac-Phe-Arg-Ala-Asn-Ser(O-Gly)-Phe-Arg-Ala

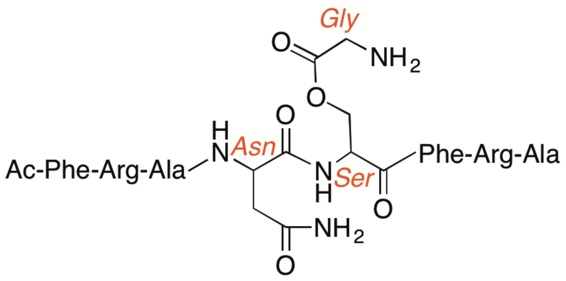

Ac-FRANS(O-Gly-H)FRA

The Fmoc chemistry stepwise SPPS protocol used was: scale 0.1 millimol H-Ala-O-2-Chlorotrityl-(S-DVB)resin; DMF washes: 10 sec flow, 1x1min batch; Fmoc-AA (0.55 mmol) dissolved in 0.5M HBTU in DMF (1 mL, 0.5 mmol); 0.75 mmol DIEA (131 μL) was added; after 30 sec activation, the solution was added to the peptide-resin; coupling 30 min. N^{α}Fmoc removal: 20% v/v piperidine/DMF 2x5min batch treatments.

After the Fmoc-Arg and Fmoc-Phe residues were added to the H-Ala-O-2-Chlorotrityl-(S-DVB)resin, the Fmoc group was removed and the Fmoc-Ser[O(Boc-Gly)]-OH dipeptide (0.35 mmol) was coupled by an HATU (0.3 mmol)/DIEA (0.6 mmol) mediated protocol in DMF for 30 min. Then the remainder of the sequence from Fmoc-Asn through Fmoc-Phe was assembled by Fmoc chemistry stepwise SPPS using the HBTU/DIEA mediated coupling protocol described above. After final N^{α}Fmoc deprotection at the N-terminus, the free amino group was acetylated using 20% Ac_2O/pyridine for 30 min. The peptide-resin was then washed with DMF followed by DCM.

Then the product peptide was cleaved from the -Ala-2-Chlorotrityl-(S-DVB)resin and simultaneously deprotected by subjecting it to TFA/TIPS/water/EDT (95:2:2:1 v/v) conditions at ambient temperature. After 3h, the cleavage mixture was collected in a RB flask and the resin was washed once again with 1mL TFA. The filtrate was evaporated and the solid residue was triturated with 1:1 ice-cold ether and hexane (2x). The resultant white precipitate was then dissolved directly in 6M GuHCl and purified using RP prep-HPLC on C18 column (9.4x250 mm) to give the target peptide in 4.1% yield (4.4 mg, 4.1 μmol).

Mass calculated for Ac-Phe-Arg-Ala-Asn-Ser(O-Gly)-Phe-Arg-Ala, 1067.1 Da (average isotope composition), Obsd. 1066.3 ± 0.1 Da.

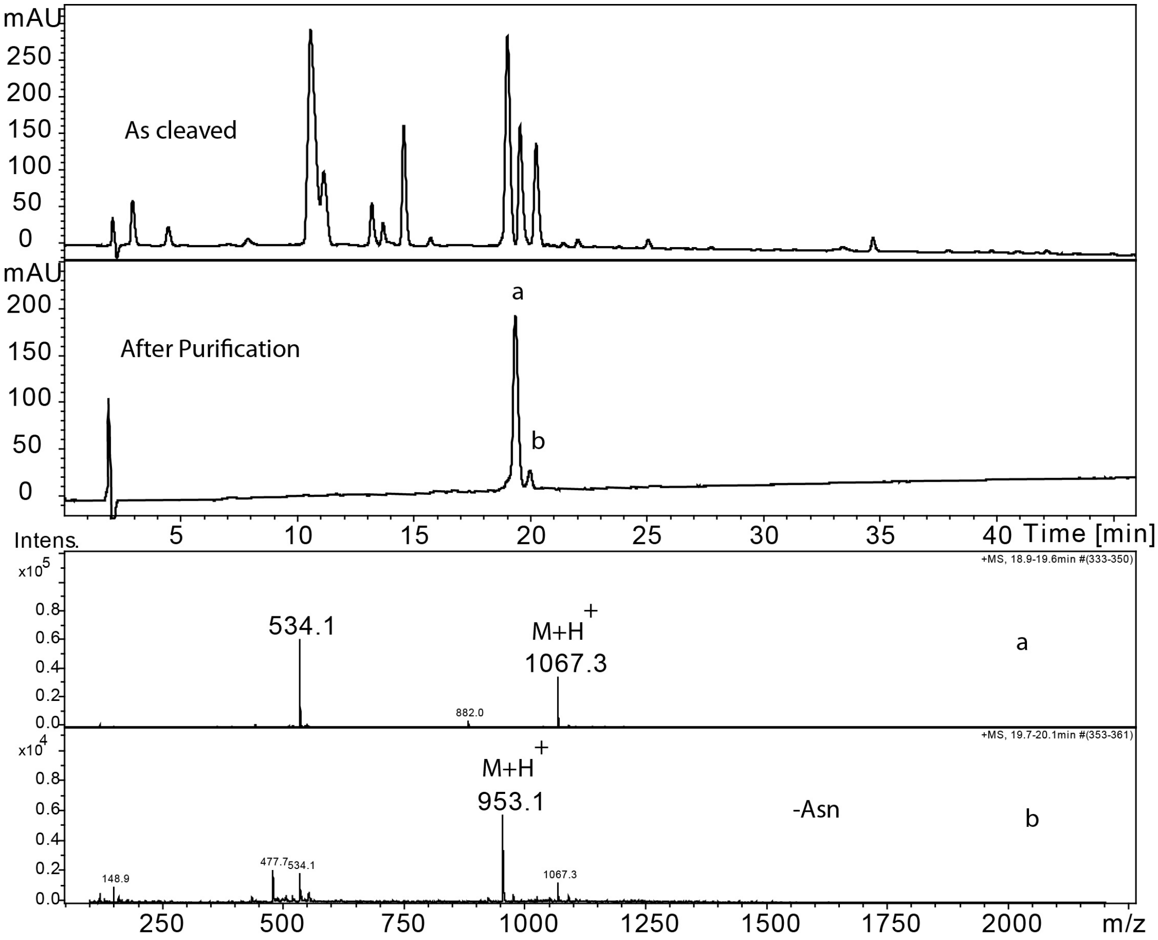

Figure S16. LC-MS data for the synthesis of Ac-Phe-Arg-Ala-Asn-Ser(O-Gly)-Phe-Arg-Ala. (Top panel) Reverse phase HPLC chromatographic separations were performed on a C8 (4.6x150 mm) column at 40 °C, using a linear gradient (1−41%) of solvent B in solvent A over 40 min (solvent A = 0.1% TFA in water, solvent B = 0.08% TFA in acetonitrile) at a flow rate of 1.0 mL/min with detection by UV absorption at 214 nm. (Bottom panel) Online ESI-MS spectrum taken across the whole of the main UV peak.

12. Synthesis of Ac-Phe-Arg-Ala-Asn-Ser(O-Gly-Ac)-Phe-Arg-Ala

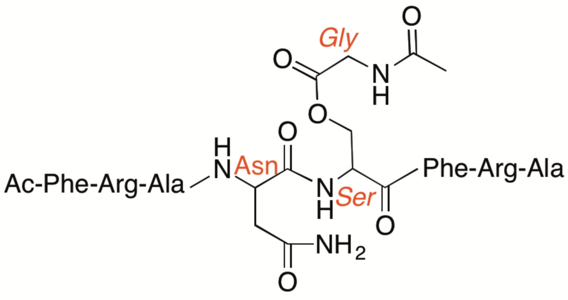

Ac-FRANS(O-Gly-Ac)FRA

Boc chemistry stepwise SPPS 'in situ neutralization' protocol: Scale Boc-Ala-OCH_2-Pam-resin (0.1 mmol). Deprotection & washing: 10 mL TFA flow (10 sec), 2x5mL TFA batch (1 min each), DMF flow (30 sec). Activation: Boc-AA-OH (0.55 mmol) was dissolved in 0.5M HBTU (0.5 mmol, 1 mL) to which was added DIEA (0.75 mmol, 131 µL); after activation for 30 sec, the solution was added to the N^{α}-deprotected peptide-resin, and the coupling was allowed to proceed for 30 min.

After addition of the Boc-Arg and Boc-Phe residues, Fmoc-Ser[O(Boc-Gly)]-OH (169 mg, 0.35 mmol) was coupled using HATU (0.3 mmol) DIEA (0.6 mmol) conditions. After removing the N^{α}-Boc group from the Gly, the revealed amino group was acetylated using Ac_2O (1.0 mmol) and DIEA (1.1 mmol). The N^{α}-Fmoc group was then removed using 20% piperidine/DMF and addition of the remainder of the residues from Boc-Asn through Boc-Phe was carried out. After removal of the final N^{α}-Boc group at the N-terminus, the free alpha-amino group was acetylated using 20% Ac_2O/pyridine for 30 min. The peptide-resin was then washed with DMF followed by DCM. Then, the peptide was cleaved from resin with simultaneous removal of all side-chain protecting groups using 5% p-cresol/HF at 0 °C for 1 h. After careful evaporation of HF at 0 °C, the resulting residue was treated with ice-cold ether. The precipitated peptide was filtered and washed with ice-cold ether (3x). Then the crude peptide was dissolved directly in 6M GuHCl and purified using RP prep-HPLC on a C18 column (9.4x250 mm) to give the target peptide in 4.2% yield (4.7 mg, 4.2 µmol). Mass calculated for Ac-Phe-Arg-Ala-Asn-Ser(O-Gly-Ac)-Phe-Arg-Ala, 1109.2 Da (average isotope composition), Obsd. 1108.4 ± 0.1 Da.

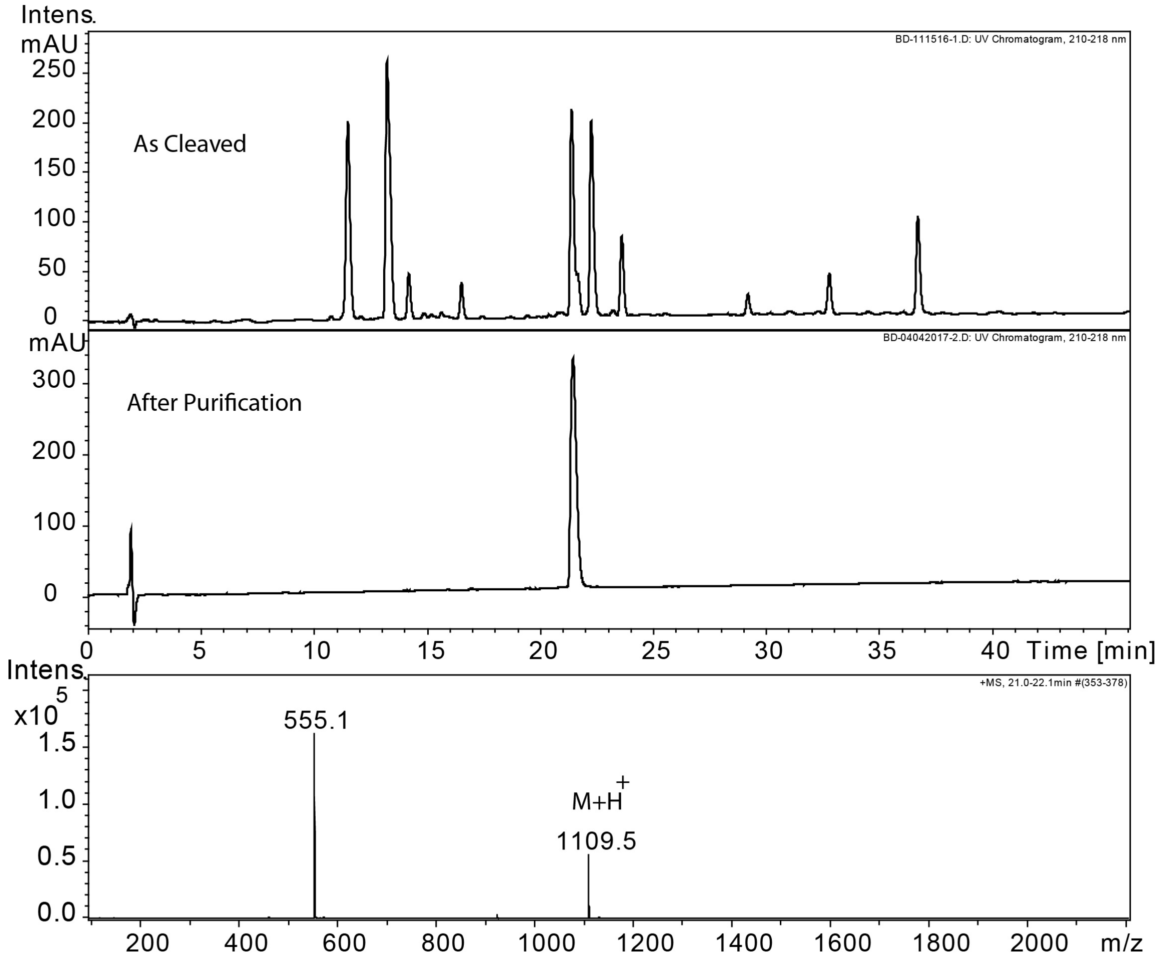

Figure S17. LC-MS data for the synthesis of Ac-Phe-Arg-Ala-Asn-Ser(O-Gly-Ac)-Phe-Arg-Ala. (Top panel) Reverse phase HPLC chromatographic separations were performed on a C8 (4.6x150 mm) column at 40 ºC, using a linear gradient (1−41%) of solvent B in solvent A over 40 min (solvent A = 0.1% TFA in water, solvent B = 0.08% TFA in acetonitrile) at a flow rate of 1.0 mL/min with detection by UV absorption at 214 nm. (Bottom panel) Online ESI-MS spectrum taken across the whole of the main UV peak.

13. Studies on Ac-FRANS(O-Gly-Ac)FRA and Ac-FRANS(O-Gly)FRA

Ac-FRANS(O-Gly-Ac)FRA (~2.3 mM) and Ac-FRANS(O-Gly)FRA (~2.3 mM) were subjected to following buffer conditions 1) *with* [6M GuHCl, 0.1 M Pi buffer, 50 mM TCEP], 2) *without* TCEP [6M GuHCl, 0.1 M Pi buffer] and three different pH values 5.5, 7.0 and 8.0 (Figures S18-S20). An aliquot was analyzed by LC-MS after acidifying to pH 3.0 at various time intervals to study the stability of these peptides.

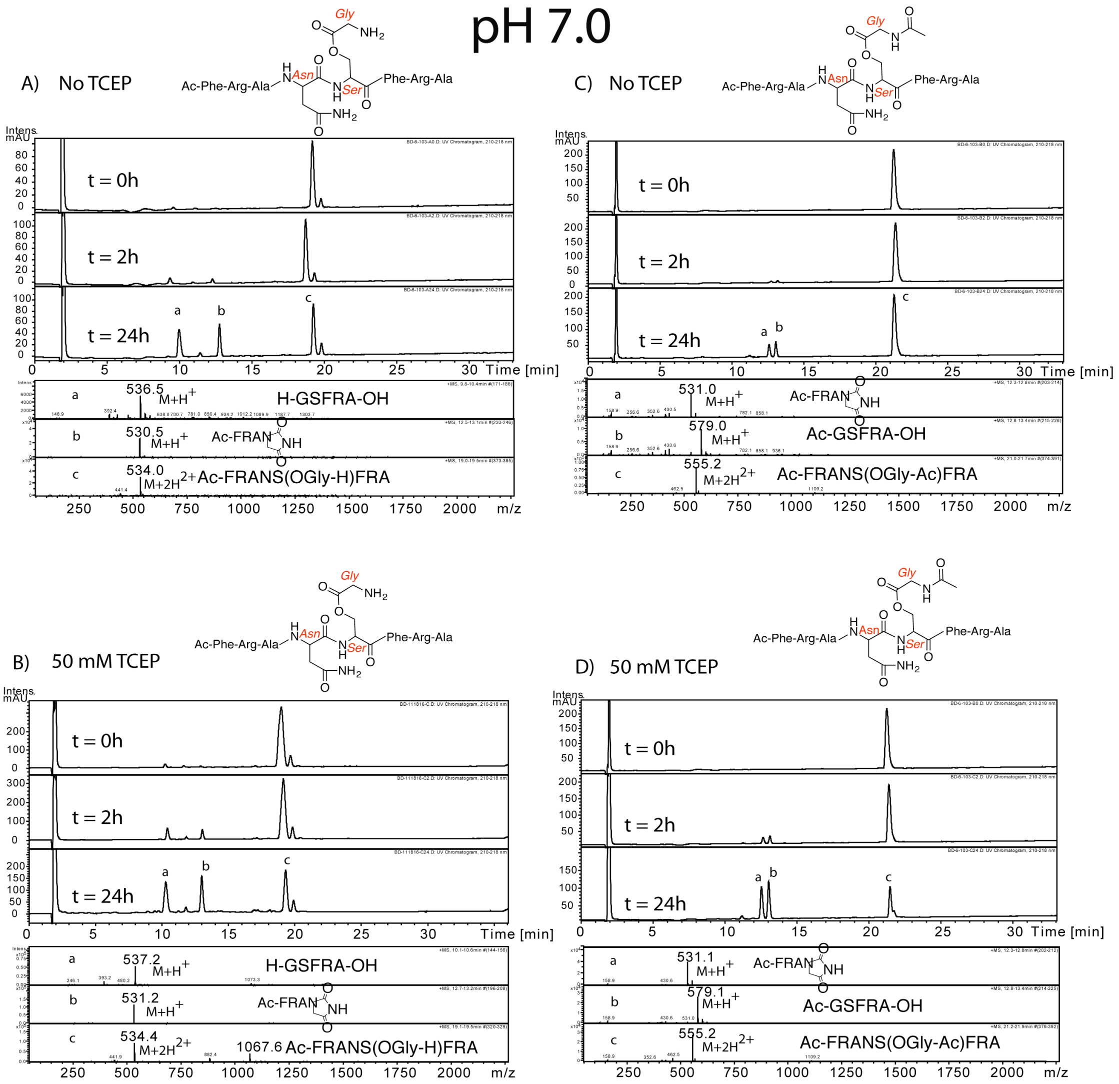

Figure S18. LC-MS data for the stability studies of Ac-FRANS(O-Gly)FRA and Ac-FRANS(O-Gly-Ac)FRA at *pH 7.0* with and without TCEP. (Top panel) Reverse phase HPLC chromatographic separations were performed on a C8 (4.6x150 mm) column at 40 °C, using a linear gradient (1−31%) of solvent B in solvent A over 30 min (solvent A = 0.1% TFA in water, solvent B = 0.08% TFA in acetonitrile) at a flow rate of 1.0 mL/min with detection by UV absorption at 214 nm. (Bottom panel) Online ESI-MS spectrum taken across the whole of the main UV peak.

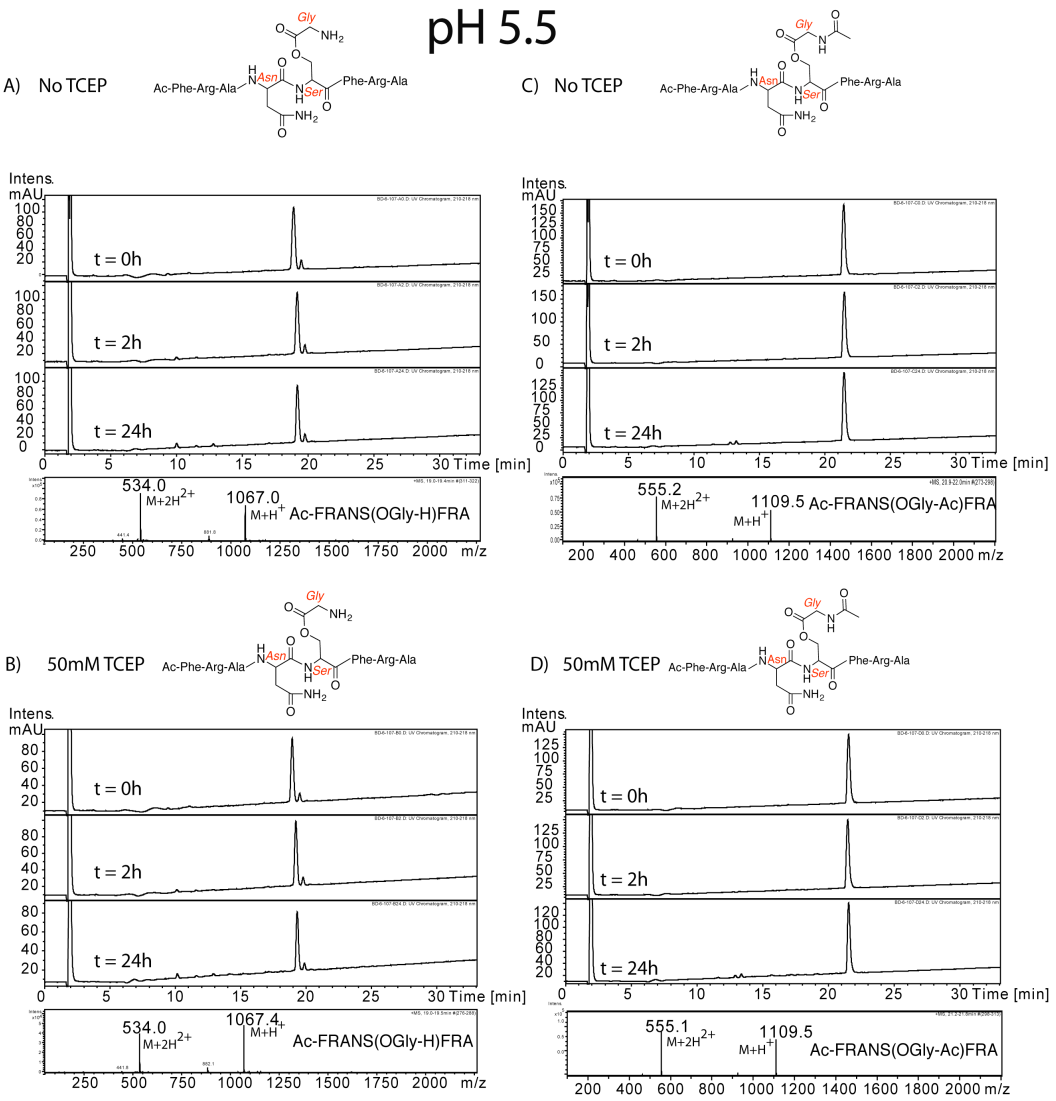

Figure S19. LC-MS data for the stability studies of Ac-FRANS(O-Gly)FRA and Ac-FRANS(O-Gly-Ac)FRA at <u>*pH 5.5*</u> with and without TCEP. (Top panel) Reverse phase HPLC chromatographic separations were performed on a C8 (4.6x150 mm) column at 40 °C, using a linear gradient (1−31%) of solvent B in solvent A over 30 min (solvent A = 0.1% TFA in water, solvent B = 0.08% TFA in acetonitrile) at a flow rate of 1.0 mL/min with detection by UV absorption at 214 nm. (Bottom panel) Online ESI-MS spectrum taken across the whole of the main UV peak.

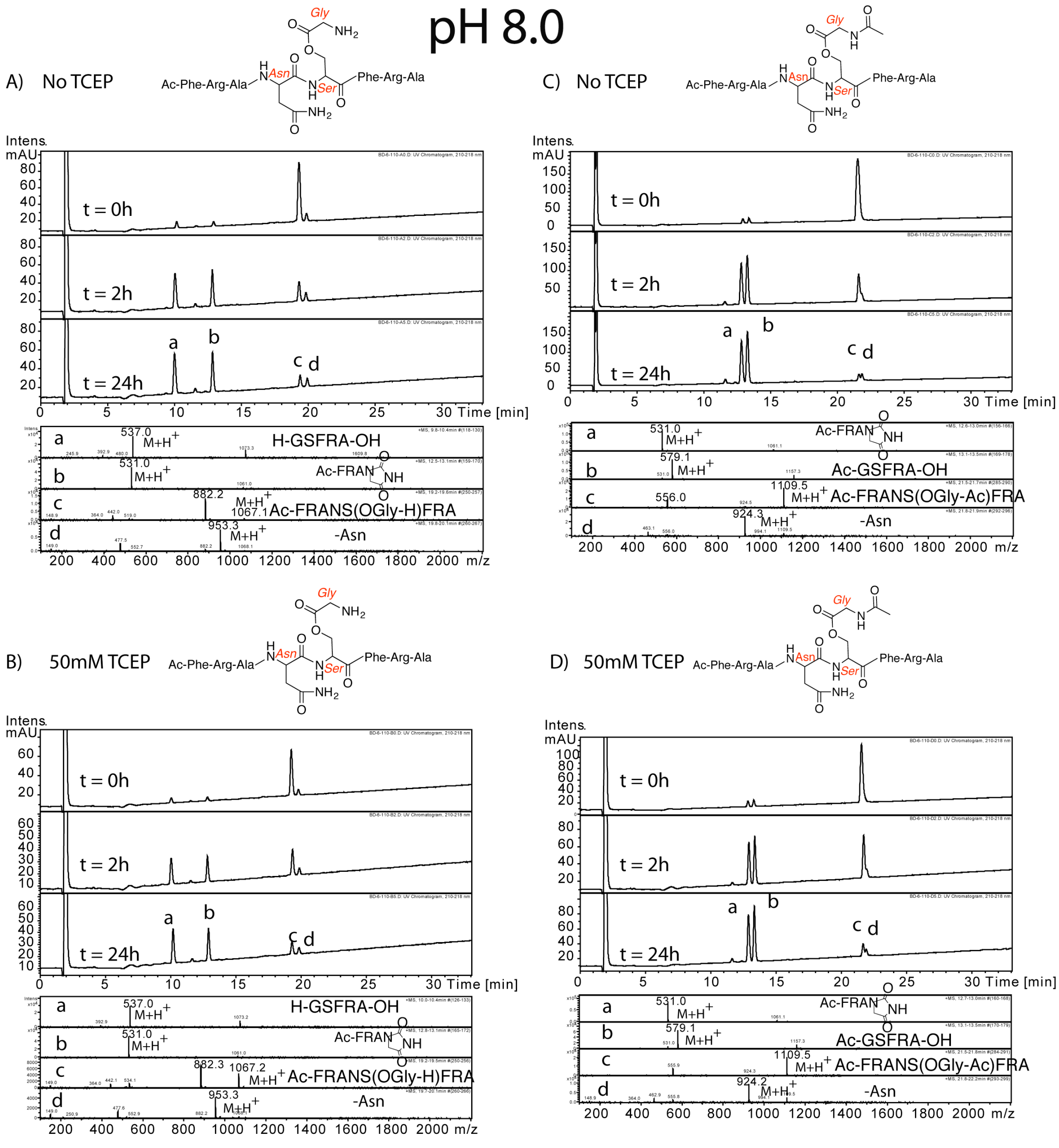

Figure S20. LC-MS data for the stability studies of Ac-FRANS(O-Gly)FRA and Ac-FRANS(O-Gly-Ac)FRA at *pH 8.0* with and without TCEP. (Top panel) Reverse phase HPLC chromatographic separations were performed on a C8 (4.6x150 mm) column at 40 °C, using a linear gradient (1−31%) of solvent B in solvent A over 30 min (solvent A = 0.1% TFA in water, solvent B = 0.08% TFA in acetonitrile) at a flow rate of 1.0 mL/min with detection by UV absorption at 214 nm. (Bottom panel) Online ESI-MS spectrum taken across the whole of the main UV peak.

14. Stability of peptide Thz^{B10}-Ser^{B28}[O(Gly^{A23}-Cys^{A22})]-Phe^{B30} at pH 7 in the presence and absence of TCEP

Peptide segment Thz^{B10}-Ser^{B28}[O(Gly^{A23}-Cys^{A22})]-Phe^{B30} was subjected to treatment under the following conditions: 1) 2.5 mM in 6M GuHCl, 0.1M Pi, 50mM Tris(2-carboxyethyl)phosphine hydrochloride (TCEP.HCl) at pH 7.0; 2) 2.5 mM in 6M GuHCl, 0.1M Pi at pH 7.0.

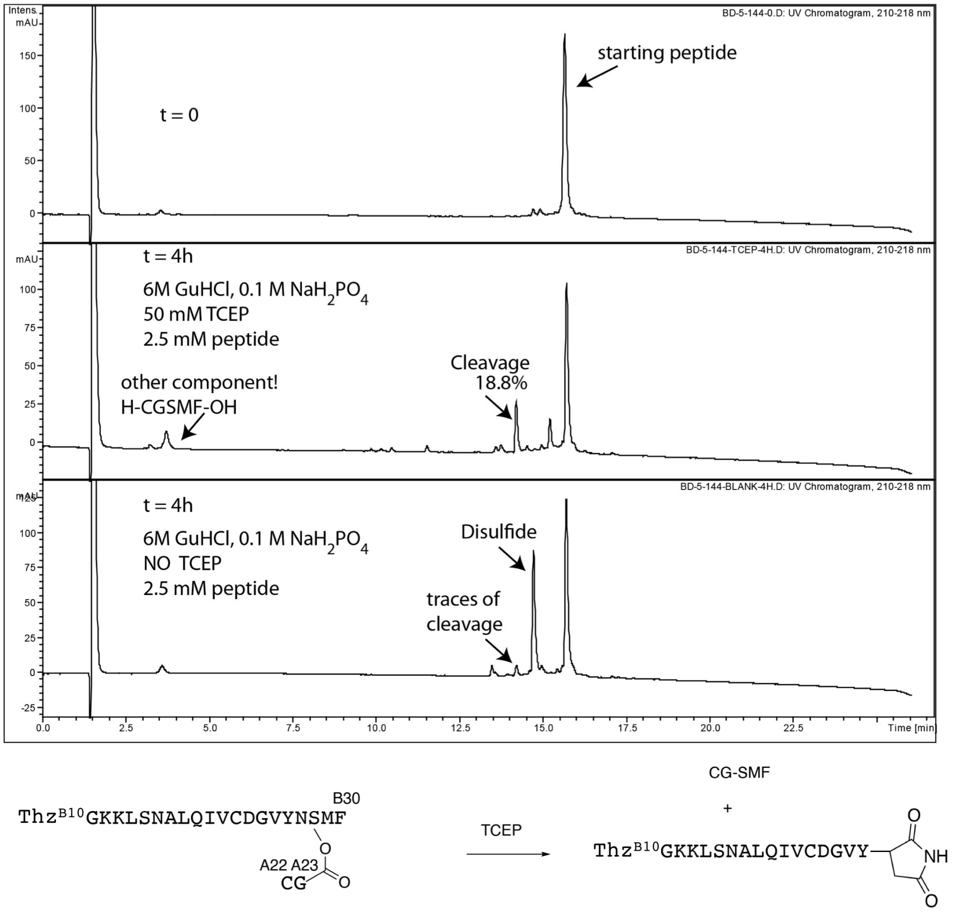

Figure S21. Cleavage of the Asn-Ser(OGly) containing peptide Thz^{B10}-Ser^{B28}[O(Gly^{A23}-Cys^{A22})]-Phe^{B30} (3) at pH 7.0 in the presence and absence of TCEP.

15. References

[1] Paul W. R. Harris , Sung Hyun Yang, Margaret A. Brimble, T*etrahedron Letter*s **2011**, *52*, 6024–6026

[2] E. C. B. Johnson, S. B. H. Kent, *Tetrahedron Lett.* **2007**, *48*, 1795-1799.

[3] B. Dhayalan, A. Fitzpatrick, K. Mandal, J. Whittaker, M. A. Weiss, A. Tokmakoff, S. B. H. Kent, ChemBioChem **2016**, *17*, 415– 420.
